# Evolutionary and Taxonomic Implications of Variation in Nuclear Genome Size: Lesson from the Grass Genus *Anthoxanthum* (Poaceae)

**DOI:** 10.1371/journal.pone.0133748

**Published:** 2015-07-24

**Authors:** Zuzana Chumová, Jana Krejčíková, Terezie Mandáková, Jan Suda, Pavel Trávníček

**Affiliations:** 1 Department of Botany, Faculty of Science, Charles University in Prague, Prague, Czech Republic; 2 Central-European Institute of Technology, Masaryk University, Brno, Czech Republic; 3 Institute of Botany, The Czech Academy of Sciences, Průhonice, Czech Republic; 4 Biotechnological Centre, Faculty of Agriculture, University of South Bohemia, České Budějovice, Czech Republic; University of Delhi, INDIA

## Abstract

The genus *Anthoxanthum* (sweet vernal grass, Poaceae) represents a taxonomically intricate polyploid complex with large phenotypic variation and its evolutionary relationships still poorly resolved. In order to get insight into the geographic distribution of ploidy levels and assess the taxonomic value of genome size data, we determined C- and Cx-values in 628 plants representing all currently recognized European species collected from 197 populations in 29 European countries. The flow cytometric estimates were supplemented by conventional chromosome counts.

In addition to diploids, we found two low (rare 3x and common 4x) and one high (~16x–18x) polyploid levels. Mean holoploid genome sizes ranged from 5.52 pg in diploid *A*. *alpinum* to 44.75 pg in highly polyploid *A*. *amarum*, while the size of monoploid genomes ranged from 2.75 pg in tetraploid *A*. *alpinum* to 9.19 pg in diploid *A*. *gracile*. In contrast to Central and Northern Europe, which harboured only limited cytological variation, a much more complex pattern of genome sizes was revealed in the Mediterranean, particularly in Corsica. Eight taxonomic groups that partly corresponded to traditionally recognized species were delimited based on genome size values and phenotypic variation. Whereas our data supported the merger of *A*. *aristatum* and *A*. *ovatum*, eastern Mediterranean populations traditionally referred to as diploid *A*. *odoratum* were shown to be cytologically distinct, and may represent a new taxon. Autopolyploid origin was suggested for 4x *A*. *alpinum*. In contrast, 4x *A*. *odoratum* seems to be an allopolyploid, based on the amounts of nuclear DNA. Intraspecific variation in genome size was observed in all recognized species, the most striking example being the *A*. *aristatum/ovatum* complex.

Altogether, our study showed that genome size can be a useful taxonomic marker in *Anthoxathum* to not only guide taxonomic decisions but also help resolve evolutionary relationships in this challenging grass genus.

## Introduction

Great strides have been made in recent years in advancing our understanding of the role of recent and ancient genome duplication in the evolution of land plants, particularly angiosperms [1–3]. The essential prerequisite for any biosystematics study dealing with ploidy-variable plant groups is the detailed knowledge of overall ploidy diversity and geographic distribution of cytotypes [4–6]. Among other information, cytogeographic data can give insight into the rate of polyploid formation, ecological differentiation of cytotypes and/or the frequency of their reproductive interactions (e.g. [7]).

In addition, because of its effects on phenotypic and reproductive traits, ploidy may serve as an important criterion guiding taxonomic delineation and may aid in interpretation of plants phylogenetic relationships [8–9].

Or perception of ploidy diversity in natural populations has been dramatically reshaped over the last two decades, after the advent and increased usage of DNA flow cytometry (FCM). This high-throughput analytical tool offers a rapid and precise method for estimating the amount of nuclear DNA across multiple populations and over large spatial scales [10–11]. Because of the rate at which samples can be processed and screened, FCM is also valuable as an exploratory tool in groups that are in need of taxonomic revision. Estimated genome size values not only allow ploidy levels to be inferred but may also provide insights into evolutionary relationships and genome constitutions of investigated species [12]. Specifically, genome size may help resolve conflicting hypotheses about the origin of polyploids (auto- vs. allopolyploidy) and identify putative parental combinations in hybridogenous species [13–18].

Genome duplication has been recognized as a driving force behind the evolutionary success of several angiosperm lineages, including Poaceae. Comparisons of diversification rates in grasses suggest that polyploidization likely led to a dramatic increase in species richness in this, the fifth largest plant family [19]. Evolutionary consequences of genome doubling in Poaceae have been extensively explored in crops (e.g. *Triticum*: [20], *Hordeum*: [21] *Oryza*: [22]), wild relatives of economically important cereals (*Brachypodium*: [23]) and weeds (e.g. *Spartina*: [24]). In contrast, the evolutionary significance of polyploidy is still little-known in many other grass genera, including *Anthoxanthum*.

*Anthoxanthum* L. (sweet vernal grass) is a relatively small genus of 15–18 species native to Europe and mountains of Asia and Africa [25–26]. At least two *Anthoxanthum* species (*A*. *odoratum and A*. *aristatum*) have been either deliberately or accidentally introduced to North America and Australia, where the species can occupy extensive areas (e.g. [27–28]). Despite the low number of recognized species, the taxonomy of the genus is non-trivial and in flux (e.g. [29]). Most traditional taxonomic treatments (e.g. [30–32]) recognize seven *Anthoxanthum* species in Europe, which can be divided into two groups according to their life forms. Whereas perennials (comprising *A*. *amarum* Brot., *A*. *alpinum* A. Löve et D. Löve, *A*. *maderense* Teppner, and *A*. *odoratum* L.) are widely distributed and show variation in ploidy levels, their annual counterparts (comprising *A*. *aristatum* Bois., *A*. *gracile* Biv., and *A*. *ovatum* Lag.) are exclusively diploid and largely restricted to the Mediterranean basin [31,33]. Several attempts have been made in recent years to clarify the taxonomy of European *Anthoxanthum* by using molecular and/or morphometric approaches [29,34–36]. These authors, among others, lumped the phenotypically similar annuals *A*. *aristatum* and *A*. *ovatum* into the *A*. *aristatum*/*ovatum* complex and stated that, based on genetic data, *A*. *alpinum* would better be regarded as a subspecies of *A*. *odoratum*.

Taxonomic complexities encountered in European members of the genus can at least partly be explained by the incidence of polyploidy. Tetraploids (2n = 4x = 20) clearly prevail in *A*. *odoratum* and rarely occur in *A*. *alpinum* [37], while *A*. *amarum* is a high-polyploid with varying numbers of somatic chromosomes (2n = 80–90; [38]). Diploid plants morphologically resembling *A*. *odoratum* (referred to as Cretan diploid [39] or diploid *A*. *odoratum* [40–41]) are known from the Mediterranean region, but their taxonomic status remains uncertain and needs to be clarified. Allozyme and karyotypic analyses [40,42–43] fairly conclusively showed that the rare tetraploids in *A*. *alpinum* are of autopolyploid origin. In contrast, there is no consensus on the origin of tetraploid *A*. *odoratum*, despite recent efforts to address this issue using molecular markers [29]. The proposed hypotheses to explain the evolutionary history of this most widespread *Anthoxanthum* species include autopolyploidization of *A*. *alpinum* [39,42], autopolyploidization of an unknown diploid Mediterranean taxon [44], or allopolyploidization involving *A*. *alpinum* and some other Mediterranean representative of the genus [40,45–46]. The origin of the highly polyploid *A*. *amarum*, endemic to the north-western part of the Iberian Peninsula [47], is enigmatic, although [35] it has been hypothesized that it likely arose by repeated polyploidization events of 4x *A*. *odoratum*.

Although the European species of *Anthoxanthum* have recently been subjected to a series of morphometric and genetic investigations (e.g., [29,34–36,48]), many questions surrounding their evolutionary history have not been satisfactorily resolved. In addition, several published papers (e.g. [29,35]) neglected karyological properties of the studied samples, which may render evolutionary interpretations problematic.

In this study, we aimed at investigating ploidy and genome size variation of the genus across its European range, and using the data to infer likely scenarios of the origin of polyploid cytotypes. Specifically, we addressed the following questions: (1) Which ploidy levels can be found among European members of *Anthoxanthum*, based on representative geographic and taxonomic sampling? (2) What is the range of genome size variation, and how does it correspond to currently recognized taxonomic groups? (3) What is the value of genome size data as a species-specific marker for taxonomic purposes and an indicator of evolutionary relationships?

## Materials and Methods

### Plant material

**Fig 1:**
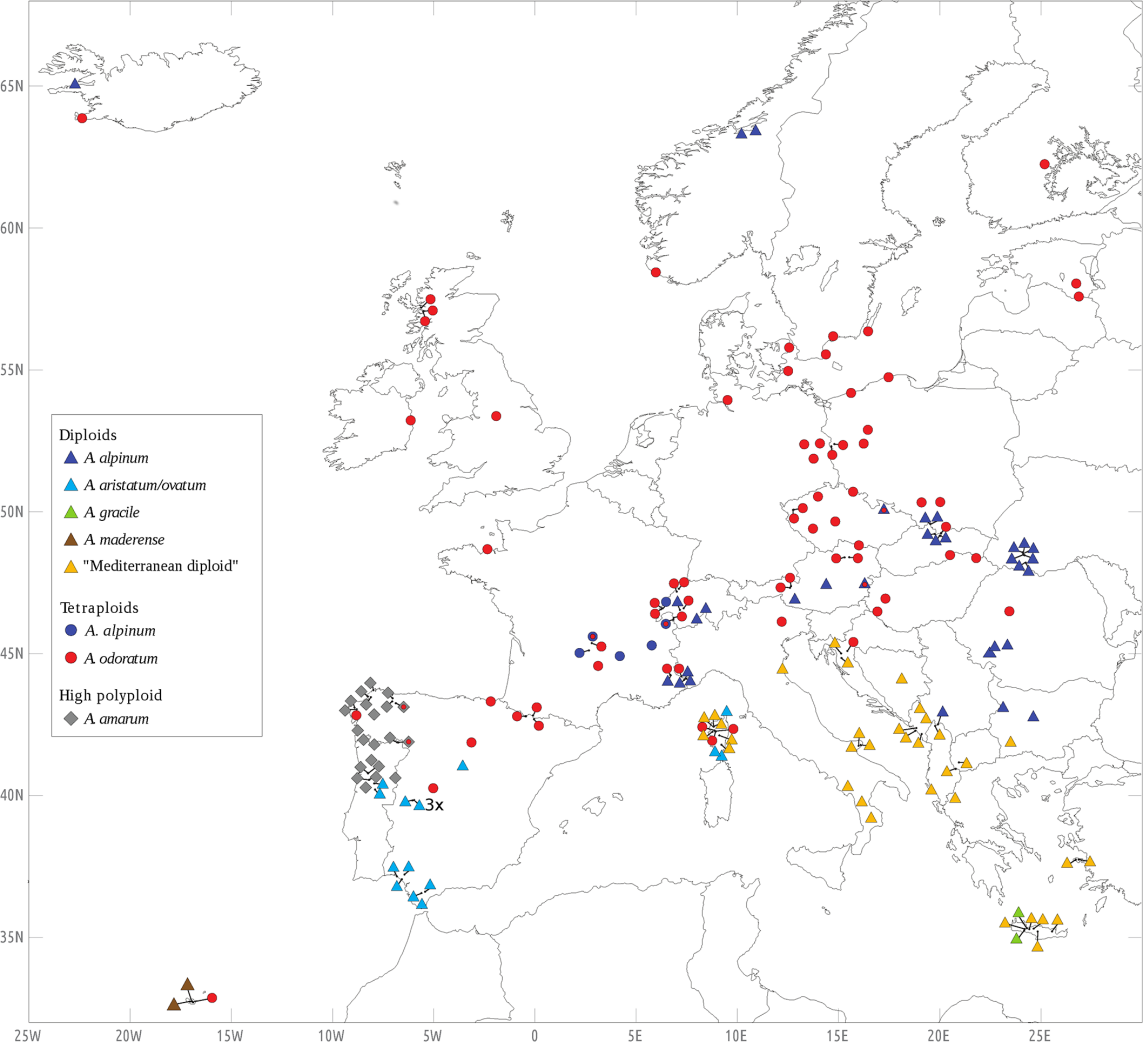
Distribution of species and cytotypes of *Anthoxanthum* in the area studied, based on analysis of 628 individuals from 197 populations sampled in 29 European countries.

A total of 197 *Anthoxanthum* populations, originating from 29 European countries were sampled during 2006–2015 (Fig 1, S1 Table). The sampling was designed to cover the entire distribution range of the genus in Europe and to include all currently recognized taxa; a major sampling effort was directed at southern Europe, which exhibits the greatest diversity [41]. At each locality, whenever possible, the following material was collected for each taxon distinguished visually: (1) flowering plants (1–17 individuals, depending on the population size) with well-developed intact leaves for FCM estimation of nuclear genome size–tissue was stored at 4°C in a plastic bag and FCM analyses were performed within a week (usually within a few days); (2) a single large tuft, for subsequent cultivation in the experimental garden of the Institute of Botany, Academy of Sciences in Průhonice, the Czech Republic (N 49°59.7´ E 14°34.0´, 315 m a.s.l.); (3) ripe caryopses from several individuals, as back-up material; (4) herbarium vouchers (deposited in the PRC).

No special permissions were required for the field study, because of (1) no endangered plants were involved in the study and (2) all localities had no protected status and were accessible by public without any restrictions.

Plants (see S1 Appendix for some scans) were identified on the basis of their morphology, following determination keys provided by [31–32,49–50]. We treated *A*. *aristatum* and *A*. *ovatum* as one species complex, based on the results of [34,36]. In addition to species appearing in the abovementioned taxonomic literature, we recognized one more group (tentatively called “Mediterranean diploid”), which we found in SE Europe and encompassed perennial diploids morphologically resembling *A*. *odoratum* occurring there. Distributional map was prepared using DMAP version 7.2e.

### Flow cytometry

Holoploid and monoploid genome sizes [51] were estimated by means of propidium iodide FCM. For each plant, one young, intact leaf, approximately 1 cm in length, was chopped along with an appropriate amount of an internal reference standard using a new razor blade in a Petri dish containing 0.5 ml of ice-cold Otto I buffer (0.1 M citric acid, 0.5% Tween 20) [52–53]. The resulting suspension was filtered through a 42-µm nylon mesh and incubated at room temperature for at least 5 min. After incubation, the suspension was stained by using 1 ml of Otto II buffer (0.4 M Na_2_HPO_4_ · 12 H_2_0) supplemented with the intercalating fluorescent dye propidium iodide, RNAase IIA (both at the final concentrations of 50 µg/ml) and β-mercaptoethanol (2 µl/ml). The samples were stained for 5 min at room temperature and analysed using a Partec CyFlow cytometer (Partec GmbH., Münster, Germany) equipped with a 532 nm diode-pumped solid-state laser Cobolt Samba (Cobolt AB, Solna, Sweden) as the source of excitation light. Fluorescence intensity of 5000 particles was recorded, and the data were analysed using Partec FloMax Software version 2.4d. *Pisum sativum* ‘Ctirad’ (2C = 8.76 pg; [53]) served as the primary reference standard. A secondary standard (*Vicia faba* ‘Inovec’, 2C = 26.60 pg) was used to analyse *A*. *amarum* and several accessions of the *A*. *aristatum*/*ovatum* complex because of large genome size and/or similarity in C-values between the sample and primary standard. Nuclear genome size of *Vicia faba* was recalibrated using *Pisum sativum*, based on repeated measurements on different days. In total, 628 *Anthoxanthum* plants were subjected to FCM analysis, and their DNA ploidy levels (*sensu* [54]) were inferred from their estimated DNA C-values, using karyologically verified plants as reference points.

### Chromosome preparations and counting

Actively growing, young roots were harvested from the cultivated plants, pre-treated with ice-cold water for 12 h, fixed in ethanol/acetic acid (3:1, v/v) fixative for 24 h at 4°C and stored at -20°C until further use. Selected root tips were rinsed in distilled water (twice for 5 min) and citrate buffer (10 mM sodium citrate, pH 4.8; twice for 5 min), and digested in 0.3% (w/v) cellulase, cytohelicase and pectolyase (all Sigma-Aldrich, St Louis, MO, USA) in citrate buffer at 37°C for 90 min. After digestion, individual root tips were dissected on a microscope slide in approximately 10 μl acetic acid and covered with a cover slip. The cell material was then spread evenly using tapping, thumb pressing and gentle flame-heating. Finally, the slide was quick frozen in liquid nitrogen and the cover slip flicked off with a razor blade. Slides were fixed in ethanol/acetic acid (3:1) and air-dried. Chromosomes were counterstained with 2 μg/ml DAPI in Vectashield (Vector Laboratories, Peterborough, UK). Preparations were analysed and photographed using an Olympus BX-61 epifluorescence microscope and CoolCube CCD camera (Metasystems, Altlussheim, Germany).

Chromosome numbers were successfully determined in all taxa but *A*. *amarum* (68 individuals from 22 populations).

### Data analysis

Genome size data were analysed using SAS 9.1.3 for Windows (SAS Institute Inc., Cary, USA). The general linear model (procedure GLM) was used to assess differences in DNA C-values among taxonomic groups, and Tukey’s test was applied to compare mean values. The Spearman-rank correlation coefficient (procedure CORR) was computed to test the association between genome sizes and geographic locations (latitude, longitude and altitude) of sampled populations.

## Results

### Ploidy variation and cytogeography

Four DNA ploidy levels were detected among the 628 analysed individuals from 197 populations: 2x, 3x, 4x, and a high polyploid with ~16–18 basic chromosome sets (Table 1). Karyological analyses of 68 individuals yielded three euploid (2n = 2x = 10, 2n = 3x = 15, and 2n = 4x = 20) and one aneuploid (2n = 16) counts, and–except for the highly polymorphic *A*. *aristatum/ovatum* complex–were fully consistent with ploidy estimates inferred from FCM measurements (Table 1, S2 Appendix). Whereas two ploidy levels occurred in *A*. *alpinum* (2x + 4x) and *A*. *aristatum/ovatum* (2x + 3x), other taxa were either exclusively diploid (*A*. *gracile*, *A*. *maderense*, and the “Mediterranean diploid”) or tetraploid (*A*. *odoratum*); the exact ploidy status of the highly polyploid *A*. *amarum* analysed in our study remains unknown. Most of the investigated populations were ploidy- and taxon-uniform, although we detected seven mixed populations, comprising three taxa combinations (Fig 1, S1 Table): (i) 2x *A*. *alpinum* + 4x *A*. *odoratum* (pops. AT03, CZ01), (ii) 4x *A*. *alpinum* + 4x *A*. *odoratum* (pops. FR05, CH05, CH06), and (iii) 4x *A*. *odoratum* + highly polyploid *A*. *amarum* (pops. PT08, ES15).

**Table 1:** Summary of recognized *Anthoxanthum* species, their ploidy levels, genome sizes (both 2C-values and 1Cx-values given in DNA picograms), intraspecific/intraploidy genome size variation and numbers of somatic chromosomes.

Life span	Taxon	DNA ploidy level	No. of individuals analysed/ No. of populations	Mean 2C-value ± s.d. [pg]	2C-value range [pg]	2C-value variation (max/min, %)	Mean 1Cx value ± s.d. [pg] *	Chromosome number (2n) / No. of individuals analysed
annuals	*A*. *aristatum/ovatum*	2x + 3x + aneuploids	147/14	7.659 ± 0.568	6.762–11.143	64.8	(3.776 ± 0.314^B^)	10/25, 15/1, 16/2
	*A*. *gracile*		5/2	18.378 ± 0.409	17.87–18.86	5.5	9.189 ± 0.205^A^	10/1
perennials	*A*. *alpinum*	2x	79/33	5.517 ± 0.083	5.361–5.692	6.2	2.759 ± 0.041^E^	10/12
		4x	20/8	10.991 ± 0.171	10.524–11.344	7.8	2.748 ± 0.043^E^	20/1
	*A*. *maderense*	2x	51/5	6.946 ± 0.094	6.795–7.378	8.6	3.473 ± 0.047^C^	10/1
	ˮMediterranean diploid“	2x	121/36	7.425 ± 0.121	7.161–7.871	9.9	3.713 ± 0.061^B^	10/14
	*A*. *odoratum*	4x	177/78	12.872 ± 0.330	12.01–13.744	14.4	3.218 ± 0.082^D^	20/11
	*A*. *amarum*	16x-18x	28/21	44.745 ± 2.173	39.513–49.74	25.9	**	-

* Different letters indicate groups of taxa that are significantly different at α = 0.05 in Tukey HSD test.

() Mean 1Cx-value for the *A*. *aristatum/ovatum* complex was calculated from 25 diploid individuals with known chromosomes number (2n = 10).

** Mean Cx-value in the highly polyploid *A*. *amarum* could not be reliably determined due to the lack of exact chromosome counts.

The most widespread cytotype across the investigated area was 4x *A*. *odoratum*, which occurred from Madeira and central Spain to Iceland and southern Scandinavia (Fig 1). The other tetraploid (4x *A*. *alpinum*) was much more geographically restricted, being found only in France and Switzerland. Whereas only two perennial species (largely diploid *A*. *alpinum* and 4x *A*. *odoratum*) occurred in the northern half of Europe, the southern part of the continent hosted much higher taxonomic diversity (and also genome size diversity, see below). Evolution in southern Europe had largely proceeded at the diploid level, with exceptions being the highly polyploid *A*. *amarum* sampled in Portugal and Spain, and rare triploids (+ aneuploids) encountered in *A*. *aristatum/ovatum* in the Iberian Peninsula (Fig 1). In addition to fairly widely distributed species, there were two narrow endemics in southern Europe, namely the perennial *A*. *maderense* (restricted to Madeira) and the little-known annual *A*. *gracile* (sampled by us only in Crete). South-Eastern Europe (the Balkan Peninsula, Crete, Italy) was dominated by the perennial taxon tentatively referred to as “Mediterranean diploid”, which there seems to replace the morphologically similar but tetraploid *A*. *odoratum*. “Mediterranean diploid” extends to Corsica where it meets 2x *A*. *aristatum/ovatum* and 4x *A*. *odoratum* (all three taxa are, however, sorted along an altitudinal gradient), making Corsica, according to our current knowledge, the region with the greatest taxonomic diversity.

### Inter- and intra-specific variation in nuclear genome size

**Fig 2:**
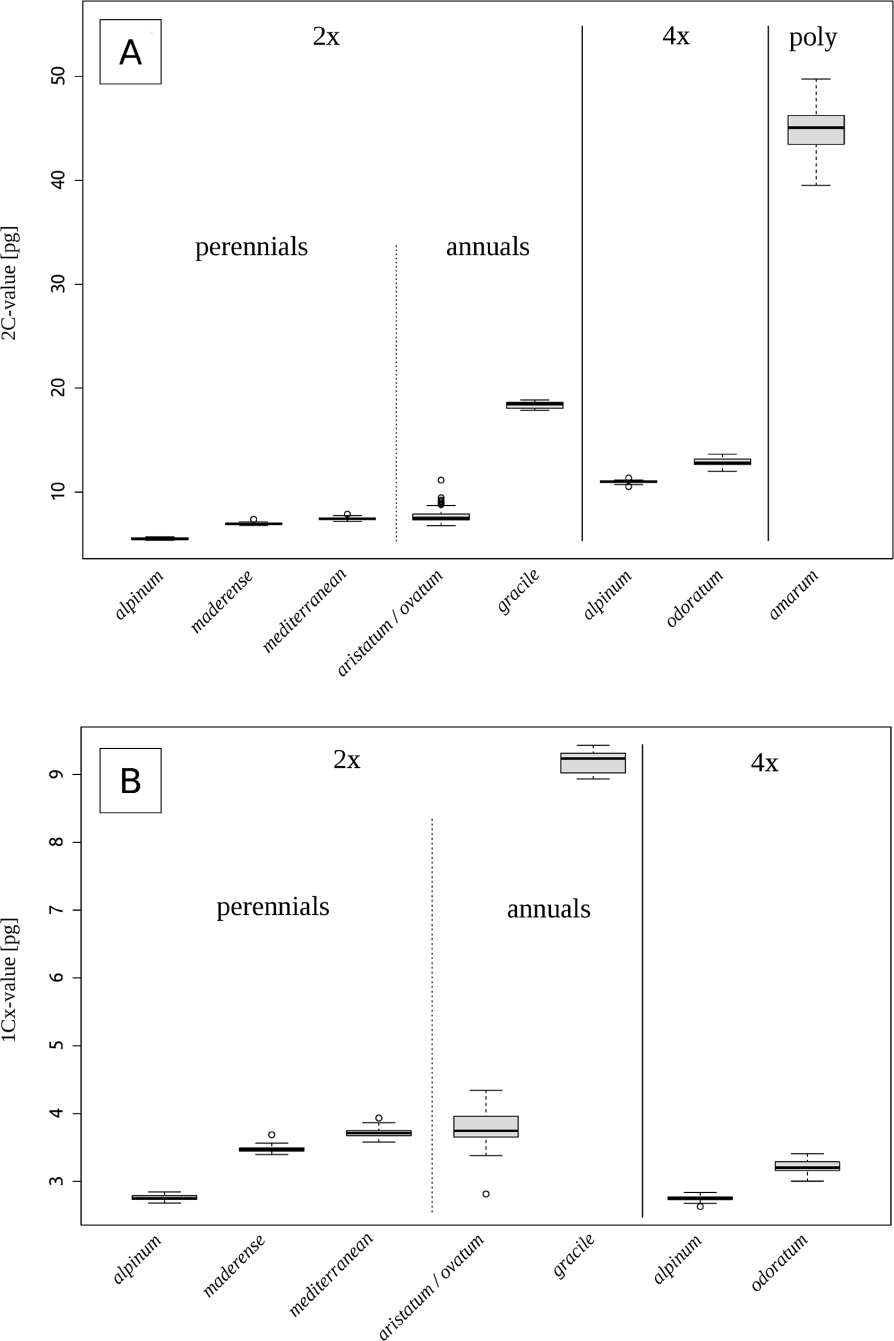
Box-and-whisker plots showing holoploid genome sizes (2C-values) for eight groups representing different species and cytotypes of *Anthoxanthum*. (A) (ploidy categories are marked as “2x”–diploids, “4x”–tetraploids and “poly”–high polyploid). (B) Box-and-whisker plots showing monoploid genome sizes (1Cx-values) for six groups representing different species and cytotypes of *Anthoxanthum* (Cx-values in the high-polyploid *A*. *amarum* and the *A*. *aristatum/ovatum* complex could not be calculated due to uncertain ploidy levels).

Mean 2C-values varied from 5.52 pg in diploid *A*. *alpinum* to 44.75 pg in highly polyploid *A*. *amarum*, spanning more than an 8-fold range (Fig 2A). Mean monoploid genome sizes (1Cx-values) ranged from 2.75 pg in tetraploid *A*. *alpinum* to 9.19 pg in diploid *A*. *gracile* (an almost 3.4-fold range, Fig 2B). (NB: 1Cx-values for highly polyploid *A*. *amarum* were not calculated, due to the lack of exact chromosome counts, which precluded reliable inference of ploidy level).

**Fig 3:**
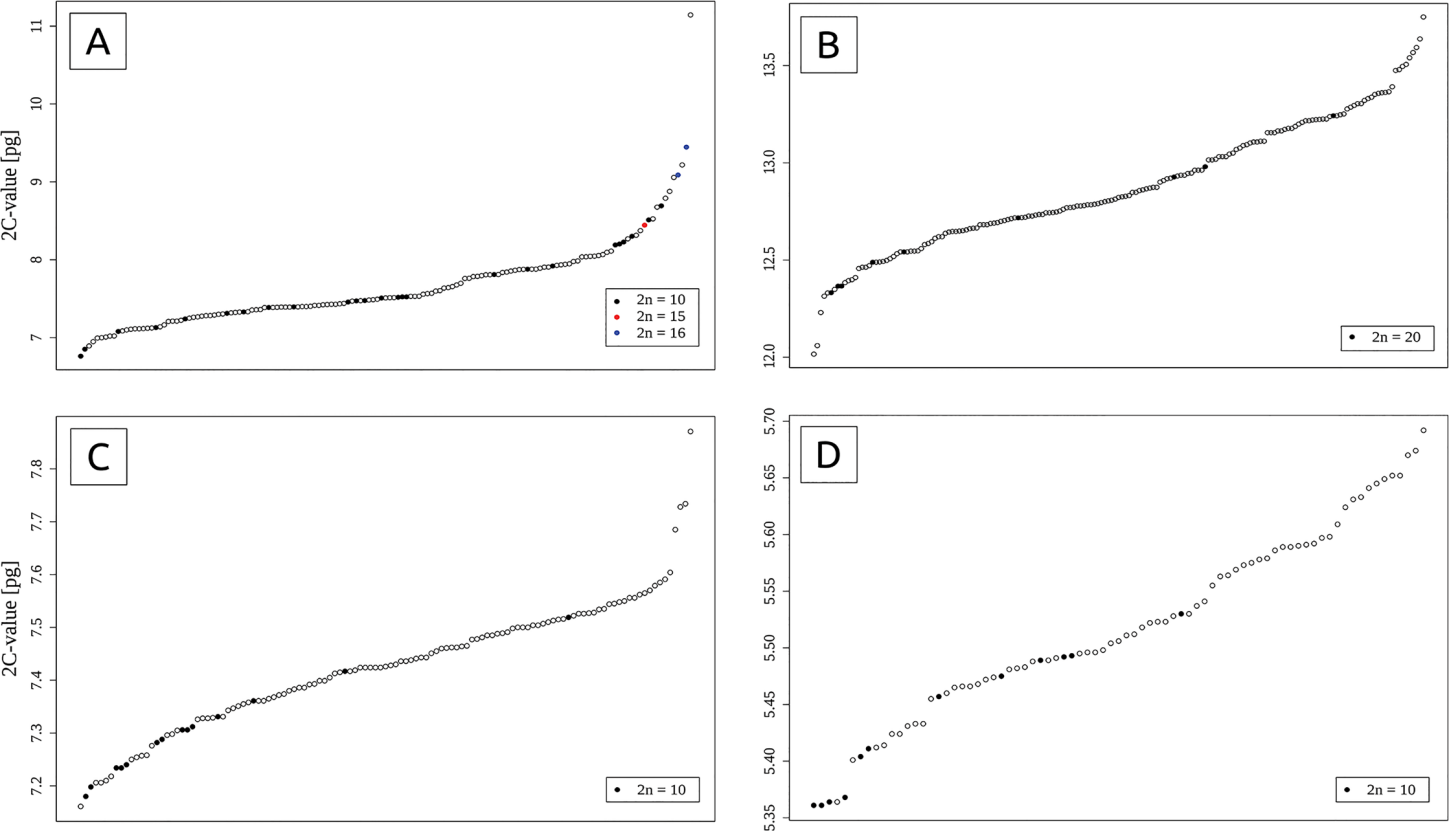
Variation in holoploid genome sizes (sorted according to increasing 2C-values) in (A) the *A*. *aristatum/ovatum* complex (total variation 64.8%); (B) 4x *A*. *odoratum* (total variation 14.4%); (C) ‘Mediterranean diploid’ (total variation 9.9%); and (D) 2x *A*. *alpinum* (total variation 6.2%). Individuals with determined numbers of somatic chromosomes are indicated by solid circles. See S1 Table for population details.

The most distinct species with respect to genome size was the annual Mediterranean endemic *A*. *gracile*, which possessed a considerably larger genome than any other European *Anthoxanthum* (Table 1). The other annual, the species complex (*A*. *aristatum/ovatum*), was excluded from statistical comparisons due to its unusually large intraspecific variation in genome size (S1 Table, Table 1, Fig 3A). All perennial taxa for which Cx-values were available differed significantly in this variable (Table 1). In fact, genome sizes of perennials formed four non-overlapping groups, arranged in ascending order as follows: *A*. *alpinum–A*. *odoratum–A*. *maderense–*“Mediterranean diploid”. Diploid and tetraploid cytotypes of *A*. *alpinum* shared very similar monoploid genome sizes, which provided support for inferring that the tetraploids are of autopolyploid origin. In contrast, Cx-values of 4x *A*. *odoratum* were distinct from those of any analysed diploid species, making autopolyploidy for this widespread tetraploid unlikely.

**Fig 4:**
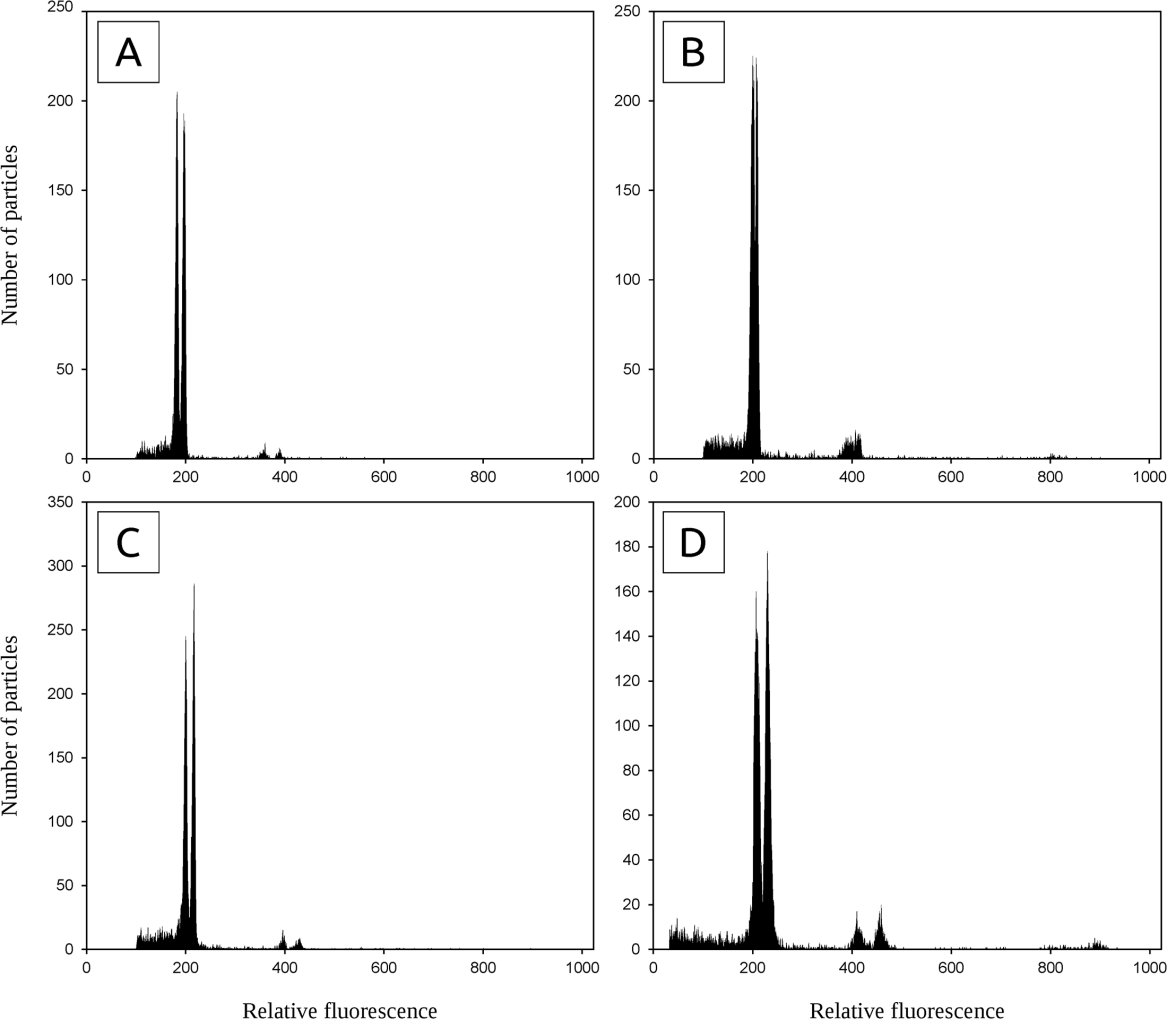
Flow cytometric histograms demonstrating genuine intraspecific variation in holoploid genome size (simultaneous analysis of individuals with distinct DNA C-values). (A) 4x *A*. *odoratum* – pops. CZ03 + HR03 (difference 8.0%); (B) ‘Mediterranean diploid’ – pops. ME05 + IT03 (difference 3.5%); (C) *A*. *amarum* – intrapopulation variation in pop. PT13 (difference 8.2%); (D) *A*. *aristatum/ovatum* – intrapopulation variation in pop. ES09 (difference 10.7%, both individuals with 2n = 10).

Intraspecific variation in genome size was observed in all analysed taxa (Table 1). 2C-values varied from 5.5% in *A*. *gracile* up to 64.8% in the polymorphic complex of *A*. *aristatum/ovatum* (Figs 3 and 4). In species collected from sufficiently large geographic areas, the intraspecific variation was non-randomly distributed and showed highly significant negative correlation with latitude (in 2x *A*. *alpinum* and 4x *A*. *odoratum*) and a less pronounced but still significant association with altitude (positive in 2x *A*. *alpinum* and negative both in the “Mediterranean diploid” and 4x *A*. *odoratum*) (Table 2, S1 Fig). Narrow geographic distribution precluded performing the same analyses for 4x *A*. *alpinum*, *A*. *gracile*, or *A*. *maderense*. *Anthoxanthum aristatum/ovatum* showed not only intraspecific but also considerable intrapopulation variation in genome size (up to 37% in pop. ES09; S1 Table, Table 1).

**Table 2:** Spearman’s correlation coefficients with corresponding p-values (in italics, significant values in bold) for population distribution data (latitude, longitude and altitude) and mean population genome sizes (2C-values) for three species with sufficiently large geographic ranges. See also S1 Fig.

Taxon	No. of populations	Latitude	Longitude	Altitude [m a.s.l.]
*A*. *alpinum* (2x)	33	**-0.775**	0.012	**0.505**
		***<0.001***	*0.915*	***<0.001***
ˮMediterranean diploid“	36	-0.021	**-0.188**	**-0.411**
		*0.820*	***0.038***	***<0.001***
*A*. *odoratum* (4x)	78	**-0.369**	-0.009	**-0.157**
		***<0.001***	*0.902*	***0.037***

## Discussion

Our study provided novel insight into ploidy and genome size variation of all currently recognized European members of the genus *Anthoxanthum*, which we sampled across large geographic areas. In addition to establishing detailed cytogeographic patterns, we also used flow cytometric and chromosome data for taxonomic and evolutionary interpretations.

### Taxonomic implications

Although the genus *Anthoxanthum* is well delimited morphologically (see [55]), its infrageneric classification is controversial due in part to phenotypic similarities of recognized species, incongruence between morphological and molecular data, and the fact that most of the published studies have been geographically limited (e.g. [34–35]). However, two groups of species are readily distinguished, based on their life cycle: annuals and perennials [55].

The most distinct perennial with respect to karyological properties is *A*. *amarum*, a highly polyploid (16x-18x) species with an unusually large genome (2C-values varying from 39.51 to 49.74 pg; Table 1) and endemic to the Iberian Peninsula. The other perennials investigated in our study possessed considerably smaller holoploid genomes (2C = 5.36–13.74 pg) and had diploid (2n = 10) or tetraploid (2n = 20) somatic chromosomes. Genome size variation was non-randomly distributed across the sampled range, and we distinguished five non-overlapping genome size groups that reflected the level of ploidy and morphology. Diploid perennials with the largest genomes (Table 1) were found in countries along the Adriatic Sea (extending eastwards up to SW Romania) and in Crete and Corsica. They very likely also occur on other Mediterranean islands not sampled in the current study (see Fig 1). These plants seem to be well delimited both geographically and karyologically, and we are convinced that they represent a distinct taxonomic entity. Traditionally, they have been identified as 2x *A*. *odoratum* [39,41–42]. However, our genome size data do not indicate close affinities with the otherwise exclusively tetraploid *A*. *odoratum*. Until the taxonomic status of large-genome perennial Balkan diploids is resolved, we refer to these plants as the “Mediterranean diploid”. Genome size of the “Mediterranean diploid” (2C = 7.16–7.87 pg) is similar to that of the putative Madeira-endemic *A*. *maderense* (2C = 6.80–7.13 pg). The third group of diploid perennial samples morphologically matched *A*. *alpinum*. This species is a near-vicariant of the “Mediterranean diploid” and has a markedly smaller genome (2C = 5.36–5.69 pg). Both taxa likely come into contact in some parts of the Balkan Peninsula (cf. Fig 1); however, the exact degree of overlap needs to be established. Considering the large differences in genome size between *A*. *alpinum* and the “Mediterranean diploid” (34% on average), FCM can be used as a reliable and rapid means to distinguish between these taxonomic groups. Our results support the view that *A*. *alpinum* is a well-defined species with unique genome size and quite distinct phenotype, despite the fact that a previous morphological and molecular study found little difference between *A*. *alpinum* and *A*. *odoratum* [29]. At the tetraploid level, there were two non-overlapping genome size categories (Table 1), corresponding to the morphologically identified *A*. *alpinum* (2C = 10.52–11.34 pg) and *A*. *odoratum* (2C = 12.01–13.74 pg). While polyploids of the former species showed a relatively narrow distribution range, 4x *A*. *odoratum* is the most widespread representative of the genus in Europe. Sympatric populations of both tetraploids were found in France and Switzerland but, reliable identification was possible based on species-specific amounts of nuclear DNA (average inter-specific difference ca. 17%).

Annual *Anthoxanthum* taxa occur only in the Mediterranean. The genome of the poorly known species *A*. *gracile*, which has mostly been reported from central and eastern Mediterranean islands [55], was markedly larger (2C = 17.87–18.86 pg) than that of the complex of *A*. *aristatum/ovatum* (2C = 6.89–11.14 pg). Although *A*. *aristatum* and *A*. *ovatum* are treated as separate species in some floras (e.g., [31,33,49]), both show striking within-species phenotypic variation and their morphological boundaries are vague [30,56]. In addition, AFLP data were unable to distinguish between *A*. *aristatum* and *A*. *ovatum* [35], and extensive introgression was reported in the Iberian Peninsula, where both taxa grow in sympatry [36]. Published data therefore support the merger of these annual sweet grasses into a highly variable diploid complex of *A*. *aristatum/ovatum* [35–36]. Unusually large and rather continuous variation in nuclear genome size (see Fig 3A), as observed in our study, provides additional evidence for treating *A*. *aristatum* and *A*. *ovatum* as a single taxonomic unit.

### Origins of polyploids

There are three different polyploid species in the genus *Anthoxanthum*: 4x *A*. *alpinum*, 4x *A*. *odoratum* and 16x-18x *A*. *amarum*.

The origin of the highly polyploid *A*. *amarum* is uncertain, although [35] presumed that it may be a derivative of 4x *A*. *odoratum*. High intraspecific variation in genome size of *A*. *amarum* (nearly 26%) detected in our study, however, precludes any firm conclusions to be made based on FCM data, and a combination of cytogenetic and molecular approaches seems to be necessary to clarify this issue.

Autopolyploid origin has been suggested for 4x *A*. *alpinum* based on karyological [40,42,45], allozyme [43] and morphological [31,56–57] evidence. Our study provides additional support for this hypothesis by revealing identical mean monoploid genome sizes of diploid (1Cx = 2.76 pg) and tetraploid (1Cx = 2.75 pg) cytotypes of *A*. *alpinum* (Table 1).

There has been a long-standing debate as to whether 4x *A*. *odoratum* originated via auto- or allopolyploidization (e.g. [29,35]). Proponents of the first hypothesis have claimed that this tetraploid is derived from either 2x *A*. *alpinum* (e.g. [58–61]) or an unknown Mediterranean diploid [44]. Assuming the additivity of genome sizes [62], we find the autopolyploid hypothesis unlikely. Resulting values for hypothetical auto-tetraploids derived from any diploid *Anthoxanthum* species differ from the actual amounts of nuclear DNA estimated for 4x *A*. *odoratum* (cf. Table 1).

In allopolyploid scenarios, 2x *A*. *alpinum* has usually been suggested as one parent [39–40,45,63]. Once again, if we accept the additive model of genome size values (see [62] for details), the most likely second parent according to our dataset is the “Mediterranean diploid” (Table 1). The sum of mean 2C-values of *A*. *alpinum* (5.52 pg) and the perennial “Mediterranean diploid” (7.42 pg) corresponds well to the average holoploid genome size of 4x *A*. *odoratum* (12.87 pg/2C). Previous karyological studies [41,45] also identified the “Mediterranean diploid” as the most promising candidate. Theoretically, our data cannot exclude the participation of *A*. *aristatum/ovatum* (suggested by [39]) or *A*. *maderense* in the genesis of 4x *A*. *odoratum*. However, the annual life cycle of *A*. *aristatum/ovatum*, narrow distribution range of *A*. *maderense*, and slightly less congruence between theoretical and actual genome size values of both taxa all speak against this possibility.

### Intraspecific variation in nuclear genome size

The issue of intraspecific variation in genome size has been of interest since the 1990s and still remains somewhat controversial. This debate has been fuelled by numerous early reports of intraspecific variation that were dismissed by subsequent investigations using the best practice methodology [64]. Over time, several sources of artefactual variation have been identified, including instrumental drift, methodological errors, disturbing effects of secondary metabolites, and taxonomic heterogeneity of the investigated material [65]. Although definitely much less common than once assumed, intraspecific variation in genome size has been recently revealed, using meticulous methodology, in several angiosperms (e.g. [5,66–69]).

In our study, we detected intraspecific variation in all recognized species (Table 1). The threshold of 10% was exceeded in three of them, namely *A*. *aristatum/ovatum*, *A*. *amarum* and *A*. *odoratum*. We argue that our FCM measurements are reliable because we strictly followed the current best practice [53], peaks with very low coefficients of variation were always achieved and, most importantly, the variation was confirmed in simultaneous analyses of samples with distinct amounts of nuclear DNA (Fig 4).

Several mechanisms are likely responsible for the observed variation in genome size in *Anthoxanthum*. At least part of the variation can be ascribed to chromosomal heterogeneity, because aneuploidy and/or the presence of supernumerary chromosomes seem to occur in most, if not all species (see S2 Table). The most variable chromosome counts (2n = 80–90) are known from a highly polyploid *A*. *amarum* [38,70], which in our study was the species with the second highest variation in genome size (25.9%).

Fairly continuous variation in genome size indicates that chromosomal heterogeneity itself cannot explain the observed pattern, and differences in the size of individual chromosomes (i.e., the genuine variation in nuclear DNA amount) must be involved. Interestingly, the variation in species with sufficiently large distribution range (2x *A*. *alpinum*, 4x *A*. *odoratum*, and the “Mediterranean diploid”) showed a clinal trend, reflecting mainly their positions along a latitudinal gradient (Table 2, S1 Fig). Because nuclear genome size of may affect several phenotypic and developmental characteristics irrespective of the information coded in the DNA (i.e., the nucleotypic effect; [71]), we speculate that the variation we found in nuclear genome size represents adaptation to different environmental conditions. Indeed, such adaptation may underlie correlations between genome size and abiotic conditions (namely latitude and altitude) found in several grass genera, including *Dactylis* [72–73], *Festuca* [67], *Koeleria* [66] and *Zea* [74].

The unusually large and continuous variation in nuclear genome size in the *A*. *aristatum/ovatum* complex was obviously caused by a combined effect of polymorphism in the number and size of somatic chromosomes, and possibly also by the complex evolutionary history of the group (e.g. introgressive hybridization; [36,48]). We found not only inter-population but also intra-population variation (reaching up to 56% in pop. FR11 and to 37% in pop. ES09, where the chromosomal counts 2n = 10, 2n = 15 and 2n = 16 were found; S1 Table) in *A*. *aristatum/ovatum*. While at least some samples of *A*. *aristatum/ovatum* with the greatest DNA amount were triploid (either euploid, 2n = 15, or euploid, 2n = 16; Table 1), large variation (28.5%) persisted even when only plants confirmed to have ten somatic chromosomes were compared (Fig 3A). Considering the maximum genome size heterogeneity reported in other angiosperms (e.g., 17% in *Festuca pallens*: [67], 18% in *Allium oleraceum*: [75], 22% in *Taraxacum stenocephalum*: [76], and 37% in *Picris hieracioides*: [68], the variation revealed in *A*. *aristatum/ovatum* is unusually large.

Despite the considerable intraspecific variation, the value of genome size as a taxonomic marker is not compromised, and all European species of *Anthoxanthum* can be identified based on the combination of life cycle (annual/perennial) and nuclear genome size (Table 1). Moreover, species with similar amounts of nuclear DNA (e.g., *A*. *maderense* and the “Mediterranean diploid”) are separated geographically.

## Supporting Information

S1 Appendix Pictures of representative plant vouchers of all species. (PDF)

S2 Appendix Micrographs of somatic metaphase chromosomes together with corresponing FCM histograms for (A-B) “Mediterranean diploid” (C-F) *Anthoxanthum aristatum/ovatum* (G-H) *Anthoxanthum gracile*. (PDF)

S1 Fig Scatter plot showing relationships between population distribution data (latitude, longitude and altitude) and holoploid genome sizes (2C-values) for three species with sufficiently large geographic ranges. (A) 2x *Anthoxanthum alpinum*; (B) “Mediterranean diploid”; (C) 4x *Anthoxanthum odoratum*.
(TIF)

S1 Table List of analysed *Anthoxanthum* populations (sorted by ploidy level and holoploid genome size for each recognized taxonomic group). For each population the information is provided about geography (population code, locality detail, coordinates in WGS-84 system and altitude), collector initials, number of analysed plants, mean holoploid genome size with standard deviation (in picograms of DNA) and intrapopulation variation (%).
(PDF)

S2 Table List of published chromosome numbers for different species of the genus *Anthoxanthum*. (PDF)

## References

[1] Soltis DE, Burleigh JG. Surviving the K-T mass extinction: New perspectives of polyploidization in angiosperms. Proc Natl Acad Sci USA. 2009;106: 5455–5456. 10.1073/pnas.0901994106 19336584 PMC2667080

[2] Symonds W, Soltis PS, Soltis DE. Dynamic polyploid formation in *Tragopogon* (Asteraceae): Recurrent formation, gene flow, and population structure. Evolution. 2010;64: 1984–2003. 10.1111/j.1558-5646.2010.00978.x 20199558

[3] Jiao YN, Wickett NJ, Ayyampalayam S, Chanderbali AS, Landherr L, Ralph PE, et al Ancestral polyploidy in seed plants and angiosperms. Nature. 2011;473: 97–100. 10.1038/nature09916 21478875

[4] Favarger C. Cytogeography and biosystematics In: Grant WF, editor. Plant biosystematics. Toronto, New York: Academic Press; 1984.

[5] Suda J, Weiss-Schneeweiss H, Tribsch A, Schneeweiss GM, Trávníček P, Schönswetter P. Complex distribution patterns of di-, tetra- and hexaploid cytotypes in the European high mountain plant *Senecio carniolicus* (*Asteraceae*). Amer J Bot. 2007;94: 1391–1401. 21636507 10.3732/ajb.94.8.1391

[6] Perný M, Kolarčik V, Majeský L, Mártonfi P. Cytogeography of the *Phleum pratense* group (Poaceae) in the Carpathians and Pannonia. Bot J Linn Soc. 2008;157: 475–485.

[7] Jersáková J, Castro S, Sonk N, Milchreit K, Schödelbauerová I, Tolasch T, et al Absence of pollinator-mediated premating barriers in mixed-ploidy populations of *Gymnadenia conopsea* s.l. (Orchidaceae). Evol Ecol. 2010;24: 1199–1218.

[8] Baack EJ. Cytotype segregation on regional and microgeographic scales in snow buttercups (*Ranunculus adoneus*, Ranunculaceae). Am J Bot. 2004;91: 1783–1788. 10.3732/ajb.91.11.1783 21652325

[9] Baack EJ, Stanton ML. Ecological factors influencing tetraploid speciation in snow buttercups (*Ranunculus adoneus*, Ranunculaceae): Niche differentiation and tetraploid establishment. Evolution. 2005;59: 1936–1944. 16261731

[10] Kron P, Suda J, Husband BC. Applications of flow cytometry to evolutionary and population biology. Annu Rev Ecol Evol Syst. 2007;38: 847–876.

[11] Suda J, Pyšek P. Flow cytometry in botanical research: introduction. Preslia. 2010; 82: 1–2.

[12] Loureiro J, Trávníček P, Rauchová J, Urfus T, Vít P, Štech M, et al The use of flow cytometry in the biosystematics, ecology and population biology of homoploid plants. Preslia. 2010;82: 3–21.

[13] Dixon CJ, Schönswetter P, Suda J, Wiedermann MM, Schneeweiss GM. Reciprocal Pleistocene origin and postglacial range formation of an allopolyploid and its sympatric ancestors (*Androsace adfinis* group, Primulaceae). Molec Phylogen Evol. 2009;50: 74–83. 10.1016/j.ympev.2008.10.009 19013534

[14] Suda J, Trávníček P, Mandák B, Berchová-Bímová K. Genome size as a marker for the recognition of invasive alien taxa in *Fallopia* section *Reynoutria* . Preslia. 2010;82: 97–106.

[15] Trávníček P, Kubátová B, Čurn V, Rauchová J, Krajníková E, Jersáková J, et al Remarkable coexistence of multiple cytotypes of the *Gymnadenia conopsea* aggregate (the fragrant orchid): evidence from flow cytometry. Ann Bot. 2011;107: 77–87. 10.1093/aob/mcq217 21059612 PMC3002475

[16] Kúr P, Štech M, Koutecký P, Trávníček P. Morphological and cytological variation in *Spergularia echinosperma* and *S*. *rubra*, and notes on potential hybridization of these two species. Preslia. 2012;84: 905–924.

[17] Mandák B, Trávníček P, Paštová L, Kořínková D. Is hybridization involved in the evolution of the *Chenopodium album* aggregate? An analysis based on chromosome counts and genome size estimation. Flora. 2012;207: 530–540.

[18] Prančl J, Kaplan Z, Trávníček P, Jarolímová V. Genome Size as a Key to Evolutionary Complex Aquatic Plants: Polyploidy and Hybridization in Callitriche (Plantaginaceae). PLoS ONE 2014;9(9): e105997 10.1371/journal.pone.0105997 25211149 PMC4161354

[19] Soltis DE, Bell CD, Kim S, Soltis PS. The origin and early evolution of the angiosperms. Ann NY Acad Sci. 2008;1133: 3–25. 10.1196/annals.1438.005 18559813

[20] Matsuoka Y. Evolution of Polyploid *Triticum* Wheats under Cultivation: The Role of Domestication, Natural Hybridization and Allopolyploid Speciation in their Diversification. Plant Cell Physiol. 2011;52: 750–764. 10.1093/pcp/pcr018 21317146

[21] Petersen G, Seberg O. On the Origin of the Tetraploid Species *Hordeum capense* and *H*. *secalinum* (Poaceae). Syst Bot. 2004;29: 862–873.

[22] Chen JF, Huang QF, Gao DY, Wang JY, Lang YS, Liu TY, et al Whole-genome sequencing of Oryza brachyantha reveals mechanisms underlying Oryza genome evolution. Nat Commun. 2013;4:1595 10.1038/ncomms2596 23481403 PMC3615480

[23] Betekthin A, Jenkins G, Hasterok R. Reconstructing the evolution of Brachypodium genomes using comparative chromosome painting. Plos ONE 2014;9(12): e115108 10.1371/journal.pone.0115108 25493646 PMC4262448

[24] Ainouche ML, Fortune PM, Salmon A, Parisod C, Grandbastien M-A, Fukunaga K, et al Hybridization, polyploidy and invasion: lessons from Spartina (Poaceae). Biol Invasions. 2009;11: 1159–1173.

[25] Clayton WD, Renvoize S. Genera Graminum: Grasses of the world. Kew Bull, Addit Ser. 1986;13: 1–389.

[26] Watson L, Dallwitz MJ. The grass genera of the world Wallingford: CAB; 1992.

[27] Allred KW, Barkworth ME. *Anthoxanthum* L In: Flora of North America 24, Barkworth ME, Capels KM & Long S, editors. Oxford University Press; 2007.

[28] Muyt A. Bush invaders of Southeast Australia RG & Richardson FJ, editors. Meredith, Victoria, Australia; 2001.

[29] Pimentel M, Sahuquillo E. Relationships between the close congeners *Anthoxanthum odoratum* and *A*. *alpinum* (Poaceae, Pooideae) assessed by morphological and molecular methods. Bot J Linn Soc. 2008;156: 237–252.

[30] Valdés B. Revisión de las especies anuales del género *Anthoxanthum* (Graminae) [Revision of the annual species of the genus *Anthoxanthum* (Graminae)]. Lagascalia. 1973;3: 99–141.

[31] Tutin TG. *Anthoxanthum* L In.: Tutin TG, Heywood VH, Burges NA, Moore DM, Valentine DH, Walters SM, Webb DA, editors. Europaea Flora, Vol. 5 Cambridge: Cambridge University Press; 1980.

[32] Teppner H. *Anthoxanthum maderense* spec. nova und *A*. *odoratum* (Poaceae-Aveneae) von Madeira und deren Chromosomen-Morphologie. Phyton. 1998;38: 307–321.

[33] Pignatti S. Anthoxanthum In.: Pignatti S, editor. Flora d'Italia, Vol. 3 Bolonia Edagricole, Italy; 1982.

[34] Pimentel M, Estévez G, Sahuquillo E. European sweet vernal grasses (*Anthoxanthum*, Poaceae; Pooideae; Aveneae): a morphometric taxonomical approach. Syst Bot. 2007;32: 43–59.

[35] Pimentel M, Sahuquillo E, Catalán P. Genetic diversity and spatial correlation patterns unravel the biogeographic history of the European sweet vernal grasses (*Anthoxanthum* L., Poaceae). Molec Phylogen Evol. 2007;44: 667–684. 10.1016/j.ympev.2007.04.006 17531509

[36] Pimentel M, Catalán P, Sahuquillo E. Morphological and molecular taxonomy of the annual diploids *Anthoxanthum aristatum* and *A*. *ovatum* (Poaceae) in the Iberian Peninsula. Evidence of introgression in natural populations. Bot J Linn Soc. 2010;164: 53–71.

[37] Felber-Girard M, Felber F, Buttler A. Habitat differentiation in a narrow hybrid zone between diploid and tetraploid *Anthoxanthum alpinum* . New Phytol. 1996;133(3): 531–540.

[38] Fernándes A, Queirós M. Contribution à la connaissance cytotaxinomique des spermatophyta du Portugal I. Gramineae [Contribution to the understanding of cytotaxonomy of angiosperms from Portugal I. Gramineae]. Bol Soc Brot. 1969;43: 20–140.

[39] Jones K. Chromosomes and the origin of *Anthoxanthum odoratum* L. Chromosoma. 1964;15: 248–274.

[40] Teppner H. Karyotypen europaischer, perennierender Sippen der Gramineen-Gattung *Anthoxanthum* . Oesterr Bot Z. 1970;118: 280–292.

[41] Felber F. Contribution à l'étude phytogéographique, biosystématique et experimentale du complexe polyploïd *Anthoxanthum odoratum* L. s. lat. [Contribution to the study of phytogeography, biosystematics and experiments in the polyploid complex of *Anthoxanthum odoratum* L. s. lat.]. Thèse à l'UNINe [PhD thesis], Neuchâtel; 1987.

[42] Hedberg I. Cytotaxonomic studies on *Anthoxanthum odoratum* L. *s*. *lat*. IV. Karyotypes, meiosis and the origin of tetraploid *A*. *odoratum* . Hereditas. 1970;48: 471–502.

[43] Zeroual-Humbert-Droz C, Felber F. Evidence from isozymes analysis of autopolyploidy in *Anthoxanthum alpinum* A. & D. Löve. Bot Helv. 1999;109: 217–227.

[44] Felber F. Sensitivity of the four cytodemes of *Anthoxanthum odoratum* l. s. Lat. (Poaceae) to Puccinia sardonensis Gäumann (Uredinales). Taxon. 1987;36(3): 573–577.

[45] Hedberg I. The genesis of tetraploid *Anthoxanthum odoratum* . Symb Bot Upsal. 1986;27: 147–154.

[46] Hedberg I. Morphological, cytotaxonomic and evolutionary studies in *Anthoxanthum odoratum* L. *s*. *lat*.–a critical review. Sommerfeltia. 1990;11: 97–107.

[47] Pimentel M, Sahuquillo E. Infraspecific variation and phylogeography of the high-polyploid Iberian endemic *Anthoxanthum amarum* Brot. (Poaceae; Pooideae) assessed by random amplified polymorphic DNA markers (RAPDs) and morphology. Bot J Linn Soc. 2007;155: 179–192.

[48] Pimentel M, Sahuquillo E, Torrecilla Z, Popp M, Catalán P, Brochmann C. Hybridization and long-distance colonization at different time scales: towards resolution of long-term controversies in the sweet vernal grasses (*Anthoxanthum*). Ann Bot. 2013;112: 1015–1030. 10.1093/aob/mct170 23912698 PMC3783235

[49] Conert HJ. Gramineae In.: Hegi G, editor. Illustrierte Flora von Mitteleuropa, I/3 Verlag Paul Parey, Berlin, Hamburg; 1988.

[50] Filipová L, Krahulec F. The transition zone of *Anthoxanthum alpinum* and *A*. *odoratum* in the Krkonoše Mts. Preslia. 2006;78: 317–330.

[51] Greilhuber J, Doležel J, Lysák MA, Bennett MD. The origin, evolution and proposed stabilization of the terms ‘Genome size’ and ‘C-value’ to describe nuclear DNA contents. Ann Bot. 2005;95: 255–260. 15596473 10.1093/aob/mci019 PMC4246724

[52] Otto F. DAPI staining of fixed cells for high-resolution flow cytometry of nuclear DNA In: Crissman HA, Darzynkiewicz Z (eds). Methods in cell biology, Vol. 33 New York, NY, Academic Press; 1990. 10.1016/s0091-679x(08)60516-6 1707478

[53] Doležel J, Greillhuber J, Suda J. Estimation of nuclear DNA content in plants using flow cytometry. Nat Protoc. 2007;2: 2233–2244. 17853881 10.1038/nprot.2007.310

[54] Suda J, Krahulcová A, Trávníček P, Krahulec F. Ploidy level versus DNA ploidy level: an appeal for consistent terminology. Taxon. 2006;55: 447–450.

[55] Clayton WD, Vorontsova MS, Harman KT, Williamson H. GrassBase–The online world grass flora. 2006 onwards. Available: http://www.kew.org/data/grasses-db.html.

[56] López-González G. Nota sobre el género *Anthoxanthum* L. (Gramineae) [Some notes on genus *Anthoxanthum* L. (Gramineae)]. Anales Jard. Bot. Madrid. 1994;51: 309–312.

[57] Pimentel M, Sahuquillo E. An approach to the study of morphological relationships among the sweet vernal grass (*Anthoxanthum* L. Poaceae, Pooideae) in the Iberian Peninsula. Bocconea. 2003;16: 731–737.

[58] Kattermann G. Über die Bildung polyvalenter Chromosomenverbande bei einigen Gramineen [About creation of polyvalent chromosome connections by some grasses]. Planta. 1931;12: 732–774.

[59] Parthasarathy N. Cytogenetical studies in Oryzeae and Phalarideae. III. Cytological studies in Phalarideae. Ann Bot. 1939;3: 43–76.

[60] Östergren G. Chromosome numbers in *Anthoxanthum* . Hereditas. 1942;33: 242–243.

[61] Hedberg I. Cytotaxonomic studies on *Anthoxanthum odoratum* L. s. lat. II. Investigations of some Swedish and of a few Swiss population samples. Symb Bot Upsal. 1967;18: 5–88.

[62] Eilam T, Anikster Y, Millet E, Manisterski J, Feldman M. Genome size in natural and synthetic autopolyploids and in a natural segmental allopolyploid of several Triticeae species. Genome. 2009;52: 275–285. 10.1139/G09-004 19234556

[63] Borrill M. Experimental studies of evolution in *Anthoxanthum* (*Gramineae*). Genetica. 1963;34: 183–210.

[64] Greilhuber J. Intraspecific variation in genome size in angiosperms: identifying its existence. Ann Bot. 2005;95: 91–98. 15596458 10.1093/aob/mci004 PMC4246709

[65] Šmarda P, Bureš P. Understanding intraspecific variation in genome size in plants. Preslia. 2010;82: 41–61.

[66] Pečinka A, Suchánková P, Lysák MA, Trávníček B, Doležel J. Nuclear DNA content variation among central European *Koeleria* taxa. Ann Bot. 2006;98: 117–122. 16698888 10.1093/aob/mcl077 PMC2803546

[67] Šmarda P & Bureš P. Intraspecific DNA content variability in *Festuca pallens* on different geographical scales and ploidy levels. Ann Bot. 2006;98: 665–78. 16868002 10.1093/aob/mcl150 PMC2803578

[68] Slovák M, Urfus T, Vít T, Marhold K. Balkan endemic *Picris hispidissima* (Compositae): morphology, DNA content and relationship to polymorphic *P*. *hieracioides* . Plant Syst Evol. 2009;278: 187–201.

[69] Trávníček P, Jersáková J, Kubátová B, Krejčíková J, Bateman RM, Lučanová M, et al Minority cytotypes in European populations of the *Gymnadenia conopsea* complex (Orchidaceae) greatly increase intraspecific and intrapopulation diversity. Ann Bot. 2012;110: 977–986. 10.1093/aob/mcs171 23002267 PMC3448425

[70] Teppner H. Poaceae in the greenhouses of the Botanic Garden of the Institute of Botany in Graz (Austria, Europe). Fritschiana. 2002;31: 1–42.

[71] Bennet MD. Nuclear DNA content and minimum generation time in herbaceous plants. Proc Roy Soc London, Ser B Biol Sci. 1972;181: 109–135. 4403285 10.1098/rspb.1972.0042

[72] Creber HMC, Davies MS, Francis D, Walker HD. Variation in DNA C-value in natural-populations of *Dactylis glomerata* L. New Phytol. 1994;128: 555–561. 10.1111/j.1469-8137.1994.tb03001.x 33874570

[73] Reeves G, Francis D, Davies MS, Rogers HJ, Hodkinson TR. Genome size is negatively correlated with altitude in natural populations of *Dactylis glomerata* . Ann Bot. 1998;82 (Suppl A): 99–105.

[74] Laurie DA, Bennett MD. Nuclear DNA content in the genera *Zea* and *Sorghum*. Intergeneric, interspecific, and intraspecific variation. Heredity. 1985;55: 307–313.

[75] Duchoslav M, Šafářová L, Jandová M. Role of adaptive and non-adaptive mechanisms forming complex patterns of genome size variation in six cytotypes of polyploid *Allium oleraceum* (Amaryllidaceae) on a continental scale. Ann Bot. 2013;111: 419–431. 10.1093/aob/mcs297 23348752 PMC3579448

[76] Trávníček P, Kirschner J, Chudáčková H, Rooks F, Štěpánek J. Substantial Genome Size Variation in *Taraxacum stenocephalum* (Asteraceae, Lactuceae). Folia Geobot. 2013;48(2): 271–284.

