# Supplementary material for: Evolutionary and Taxonomic Implications of Variation in Nuclear Genome Size: Lesson from the Grass Genus Anthoxanthum (Poaceae)
Source: PLoS One. 2015 Jul 24;10(7):e0133748. doi: 10.1371/journal.pone.0133748 (PMC4514812; doi:10.1371/journal.pone.0133748)
Supplement: S1 Appendix — (PDF) [file pone.0133748.s001.pdf]

Appendix S1. (A-L) Pictures of representative plant vouchers of all species.

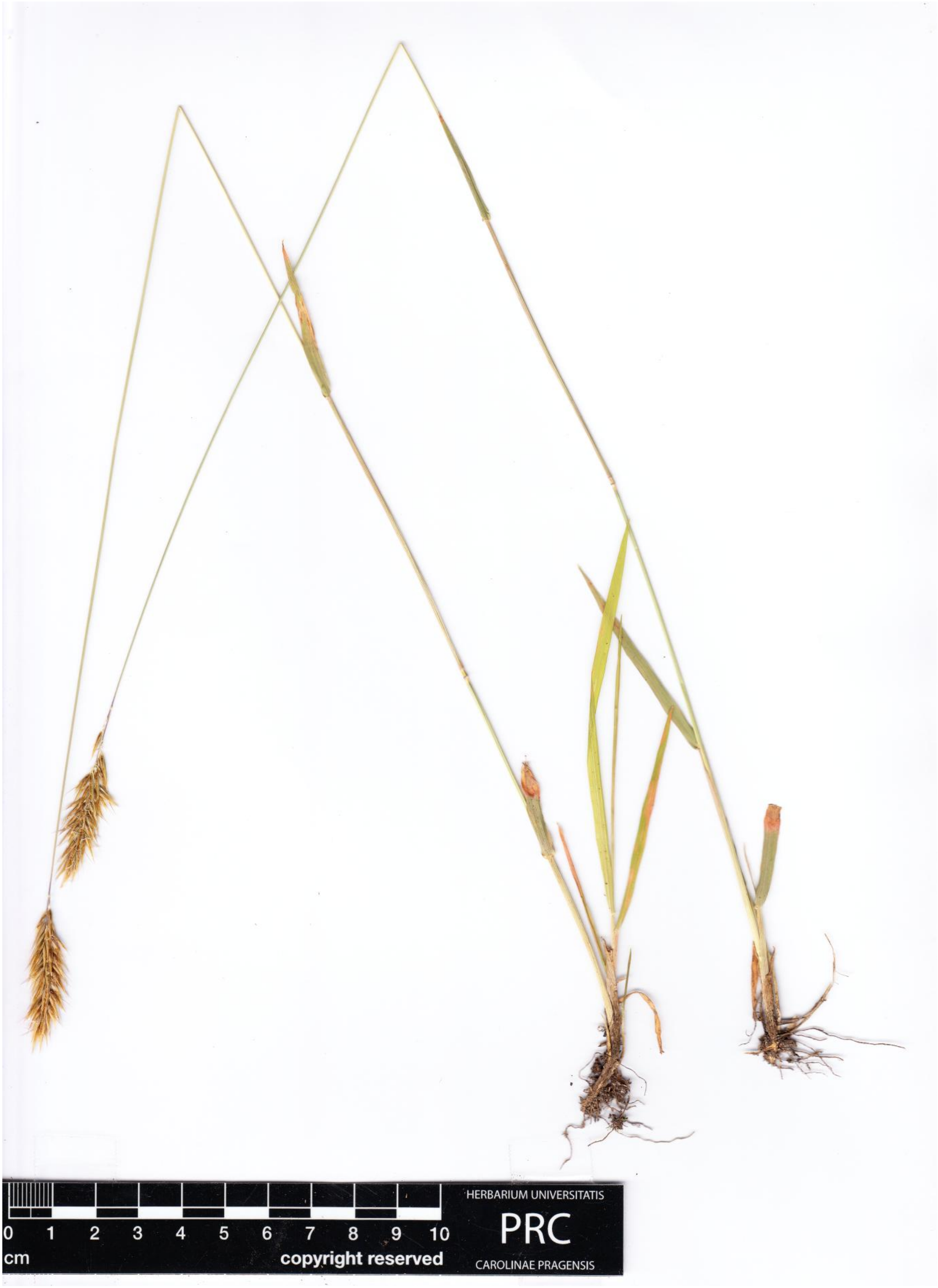

A. *Anthoxanthum alpinum*, population BG01.

B. *Anthoxanthum maderense*, population PT05.

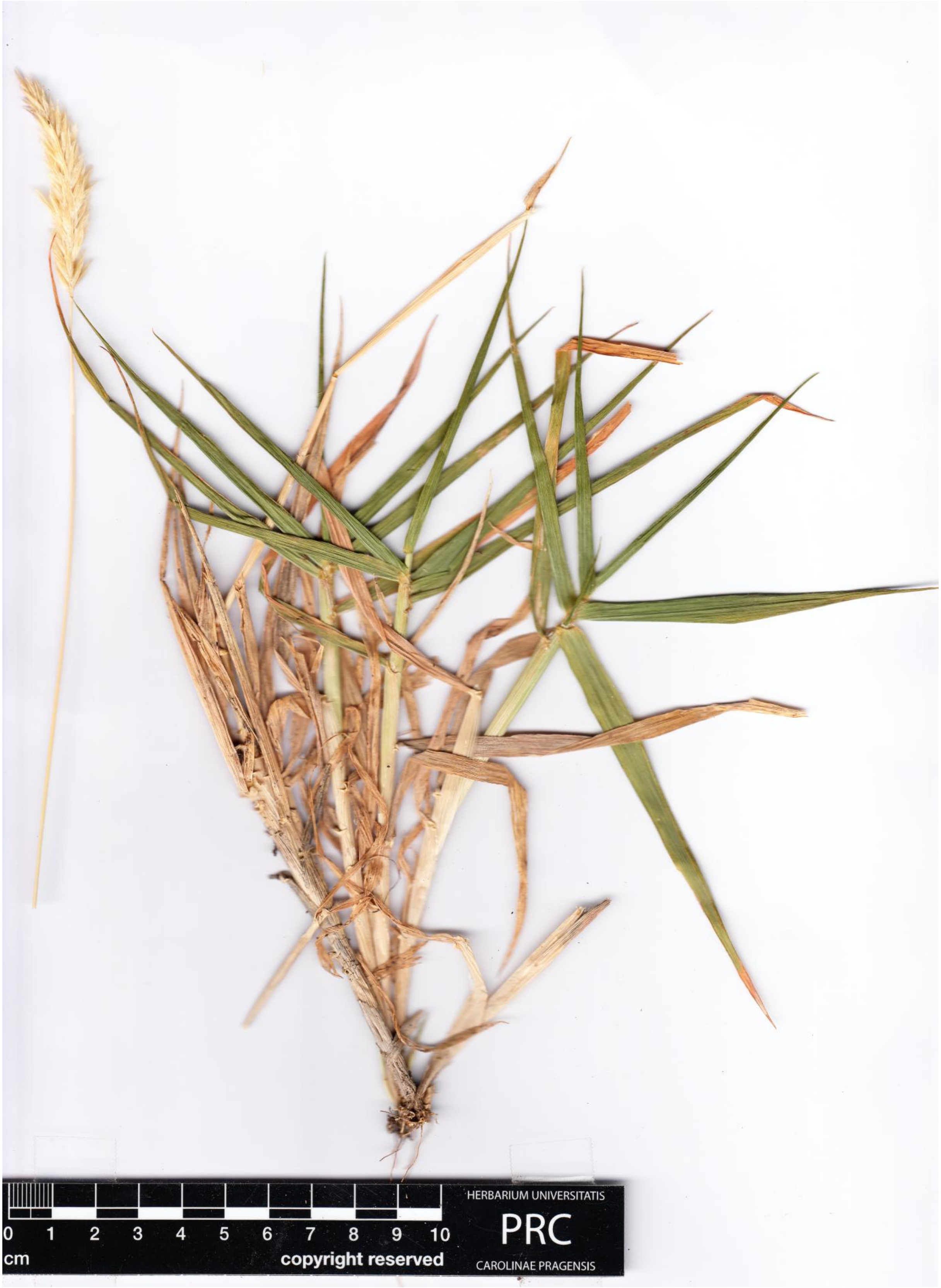

B. *Anthoxanthum maderense*, population PT05.

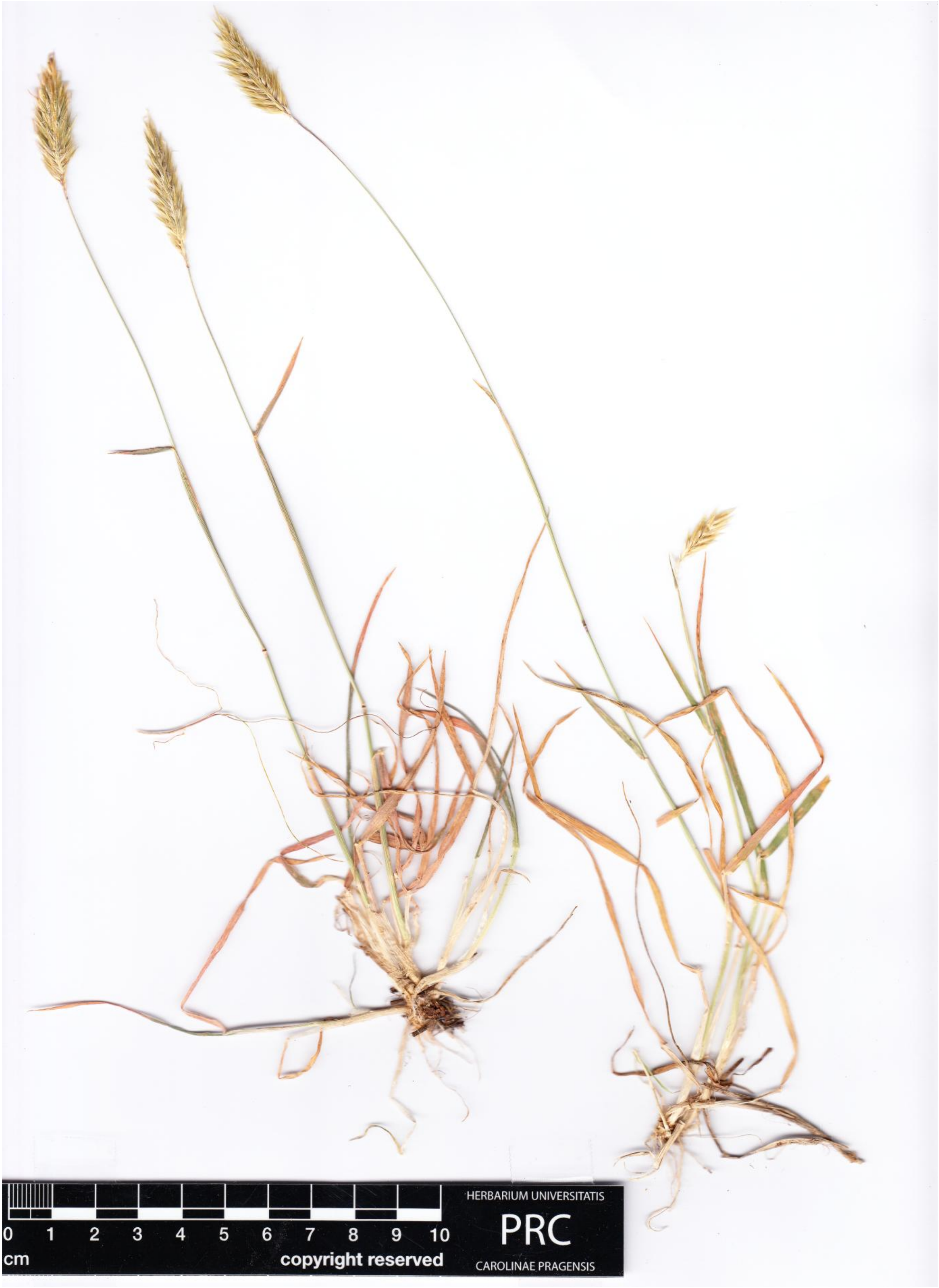

C. “Mediterranean diploid”, population GR05.

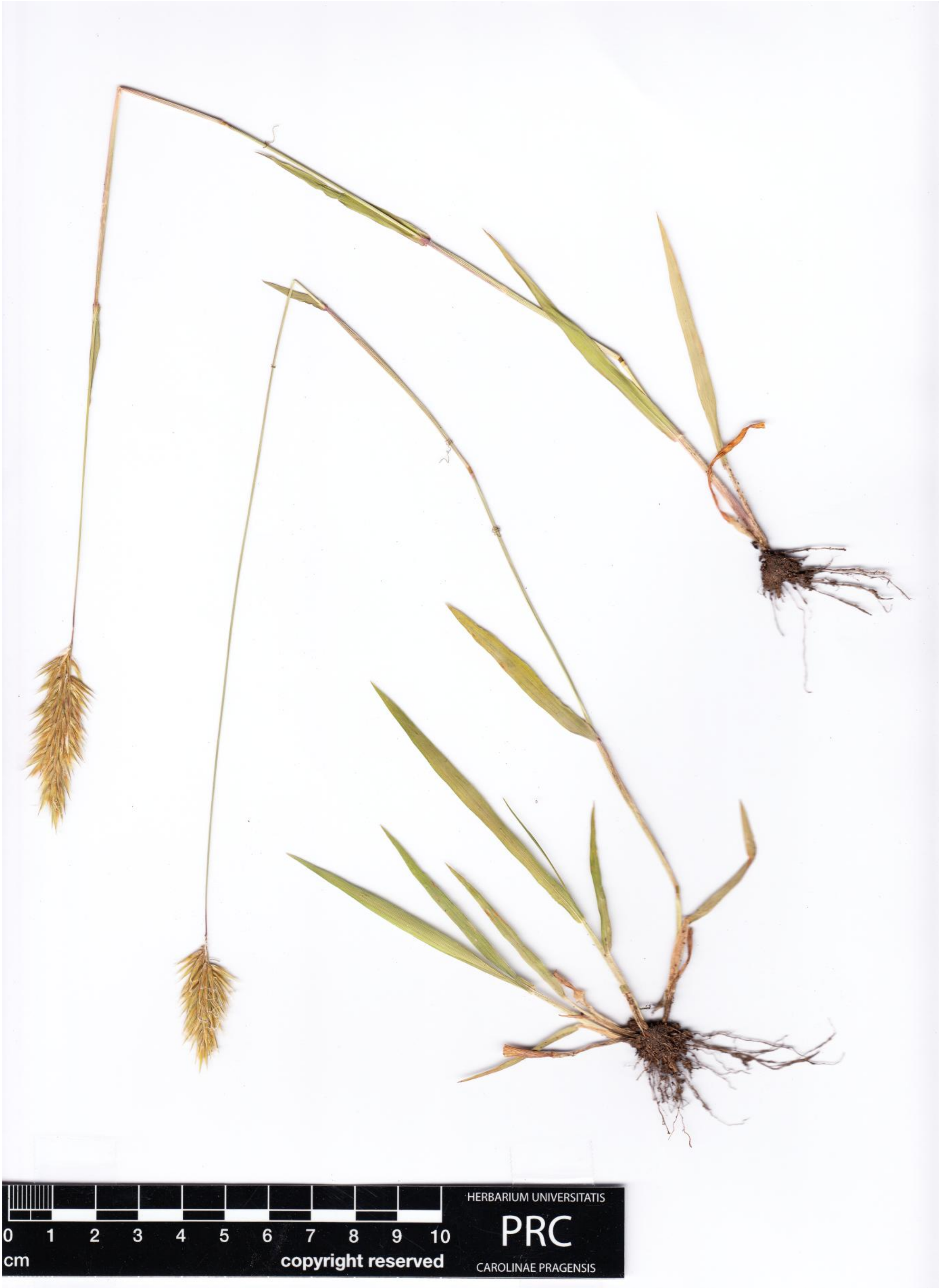

D. “Mediterranean diploid”, population IT01.

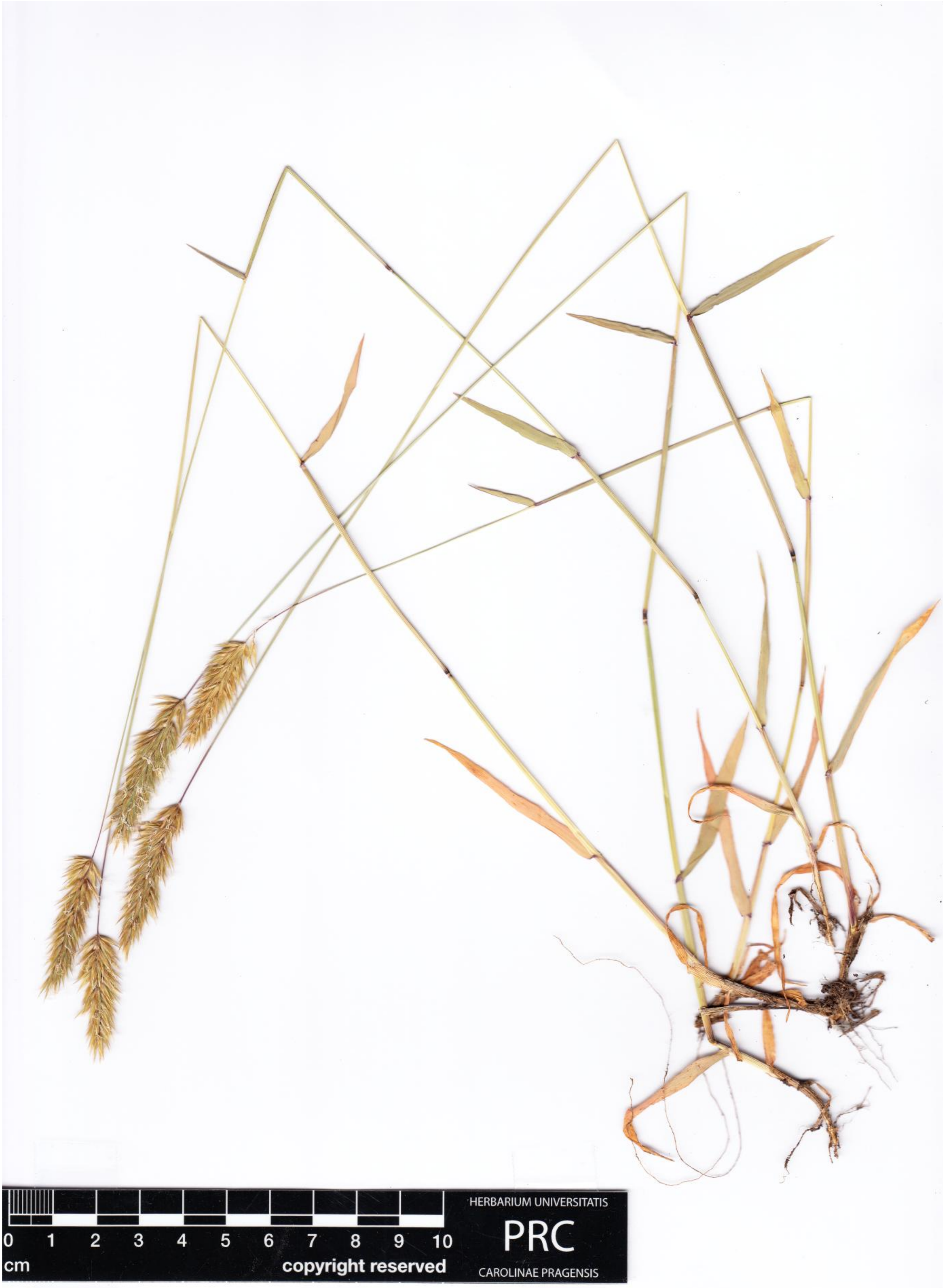

E. “Mediterranean diploid”, population ME02.

F. *Anthoxanthum aristatum/ovatum*, population ES06, plant n° 1.

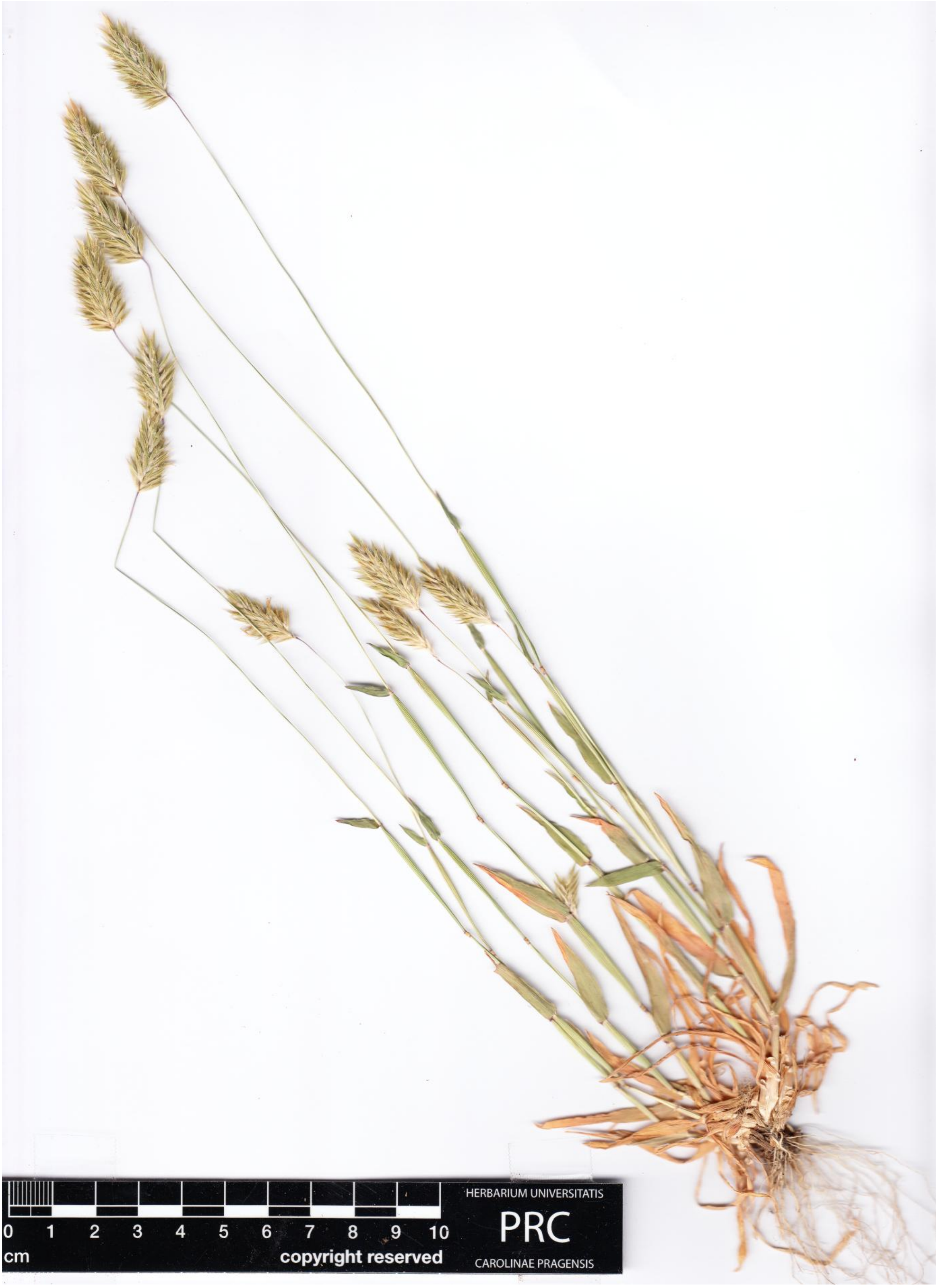

G. *Anthoxanthum aristatum/ovatum*, population ES06, plant n° 2.

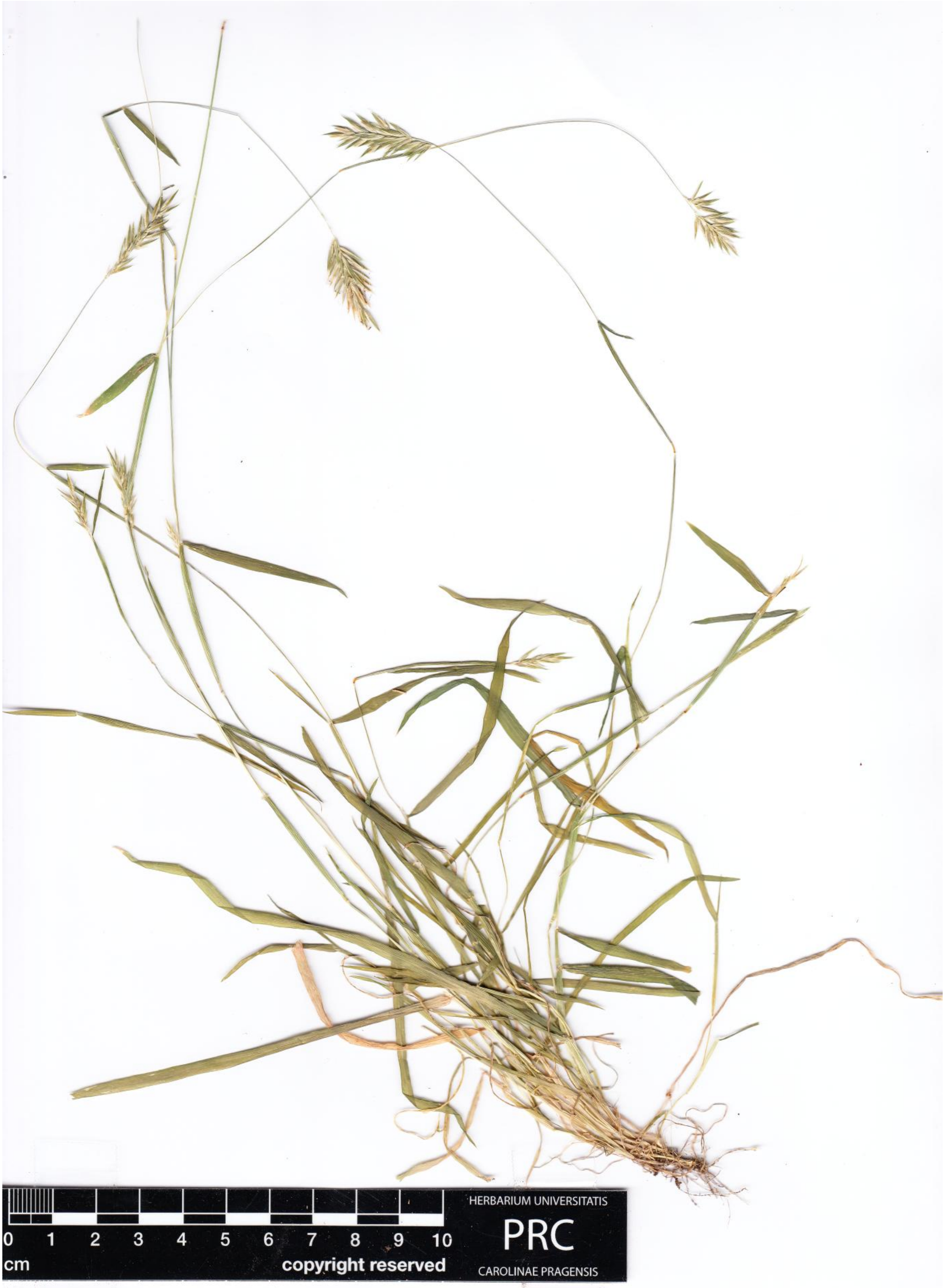

G. *Anthoxanthum aristatum/ovatum*, population ES06, plant n° 2.

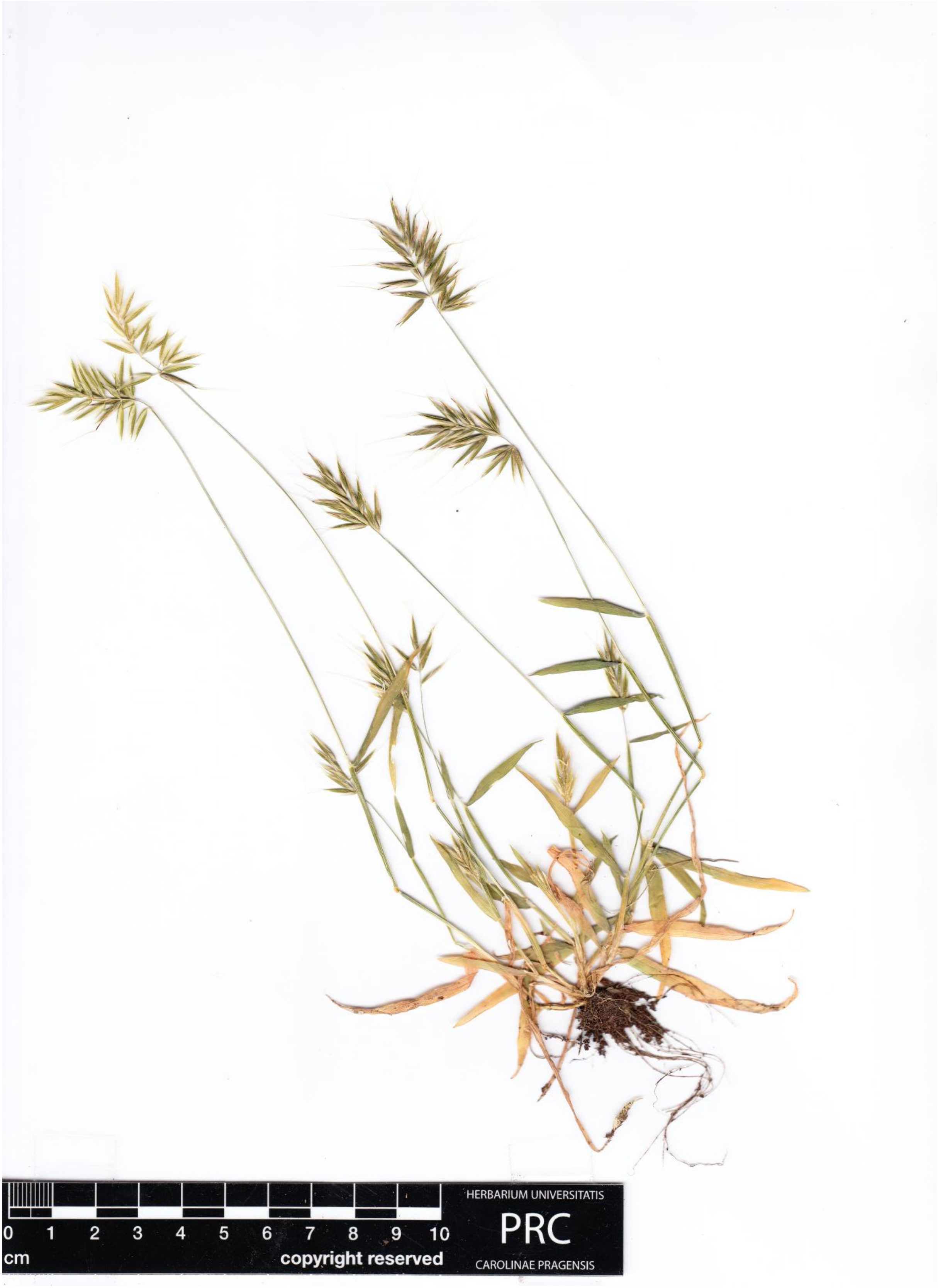

H. *Anthoxanthum aristatum/ovatum*, population ES06, plant n° 3.

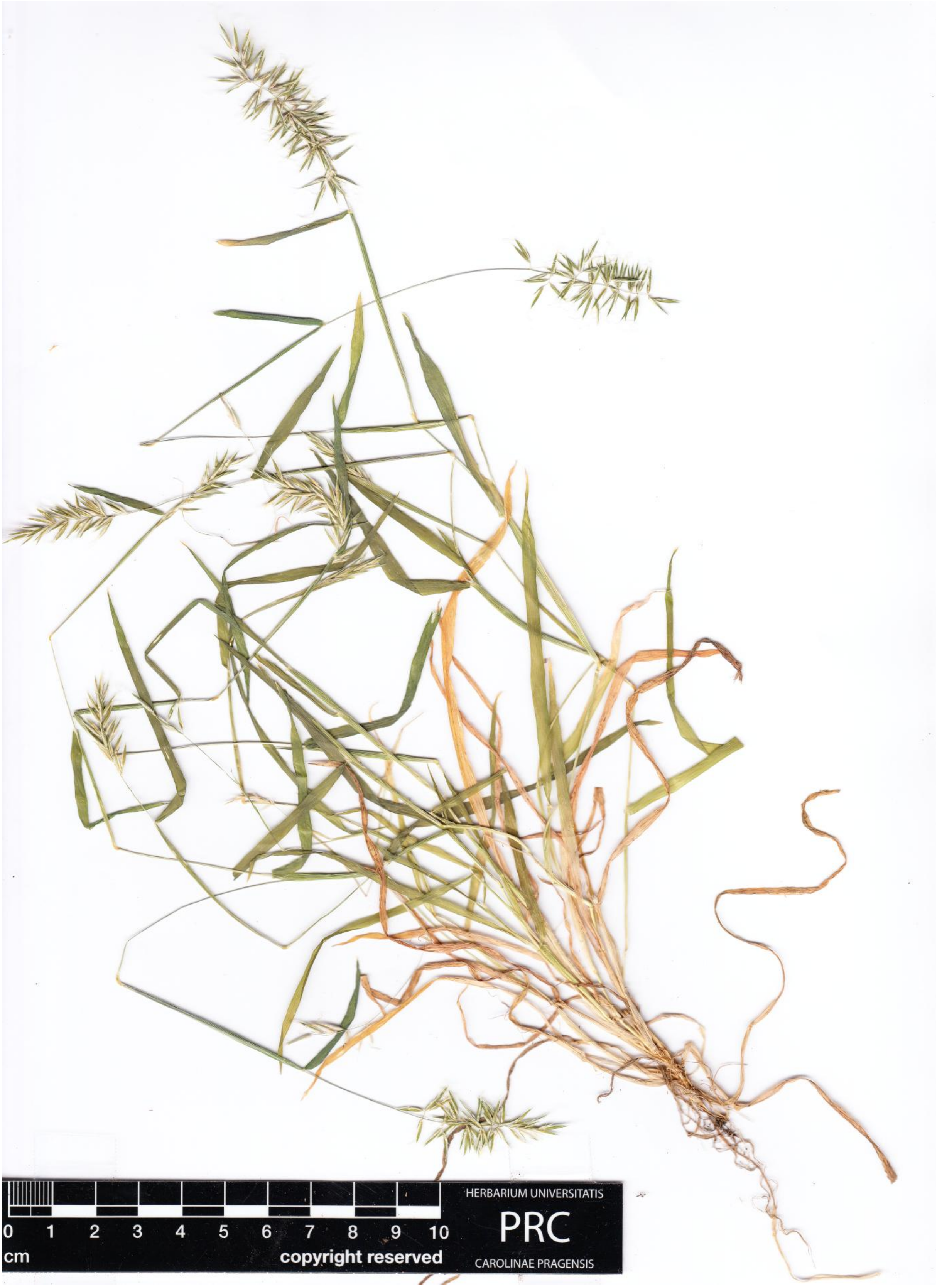

I. *Anthoxanthum aristatum/ovatum*, population ES06, plant n° 4.

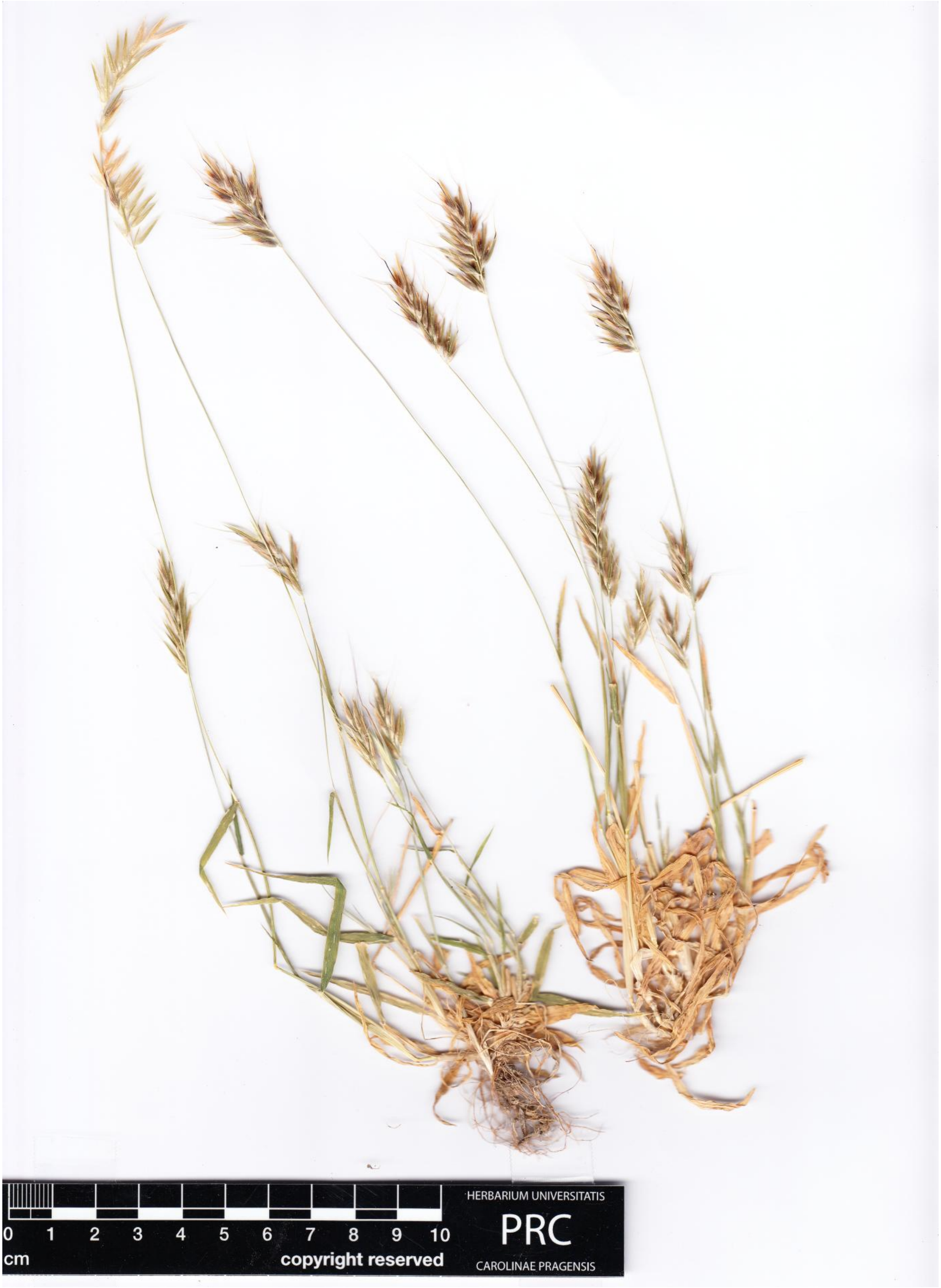

J. *Anthoxanthum gracile*, population GR09.

K. *Anthoxanthum odoratum*, population GB01.

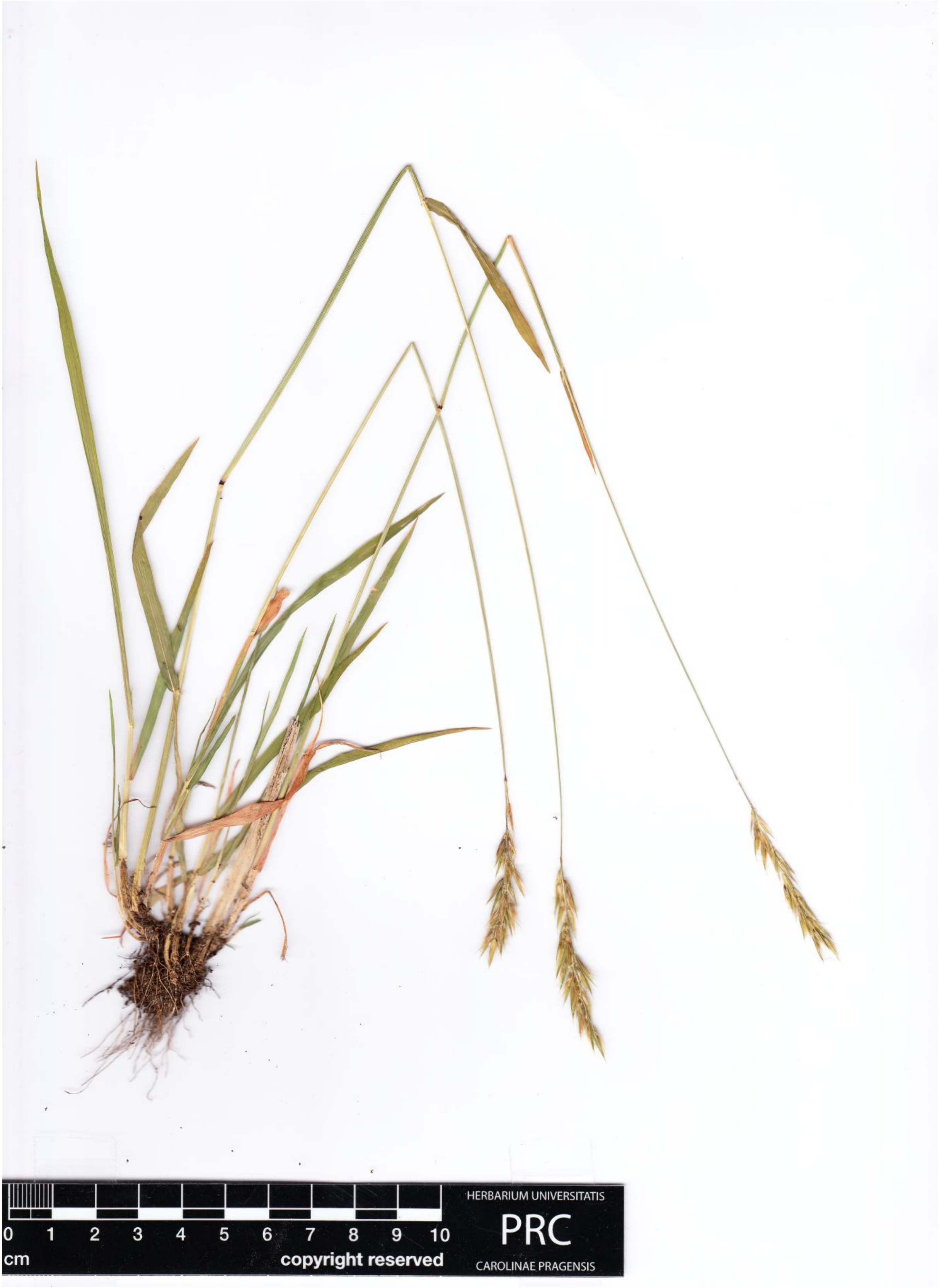

K. *Anthoxanthum odoratum*, population GB01.

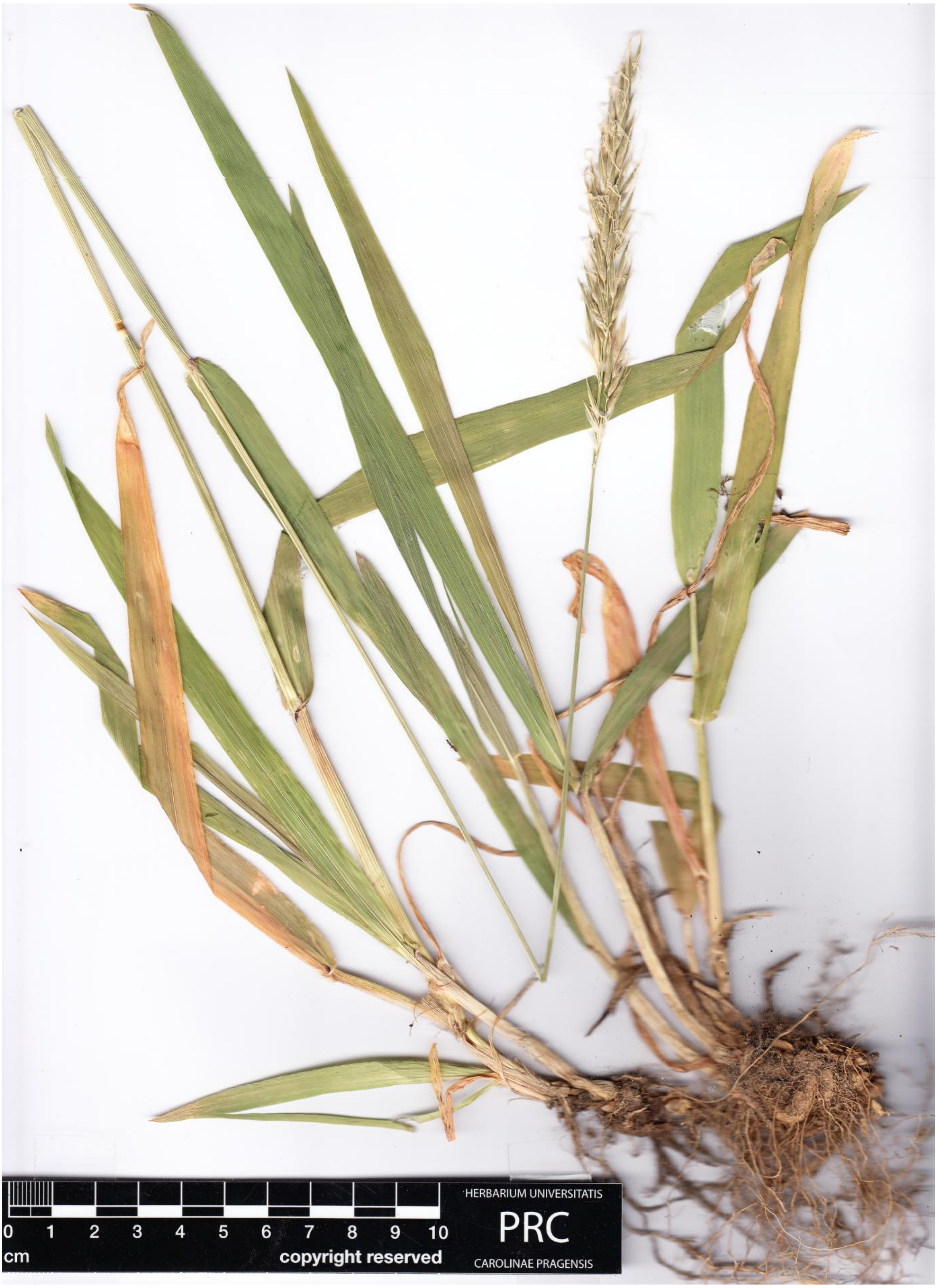

L. *Anthoxanthum amarum*, population PT12.
