# Supplementary material for: Evolutionary and Taxonomic Implications of Variation in Nuclear Genome Size: Lesson from the Grass Genus Anthoxanthum (Poaceae)
Source: PLoS One. 2015 Jul 24;10(7):e0133748. doi: 10.1371/journal.pone.0133748 (PMC4514812; doi:10.1371/journal.pone.0133748)
Supplement: S2 Appendix — (PDF) [file pone.0133748.s002.pdf]

Appendix S2. Micrographs of somatic metaphase chromosomes together with corresponding FCM histograms for (A-B) "Mediterranean diploid", (C-F) *Anthoxanthum aristatum* / *ovatum*, and (G-H) *Anthoxanthum gracile*.

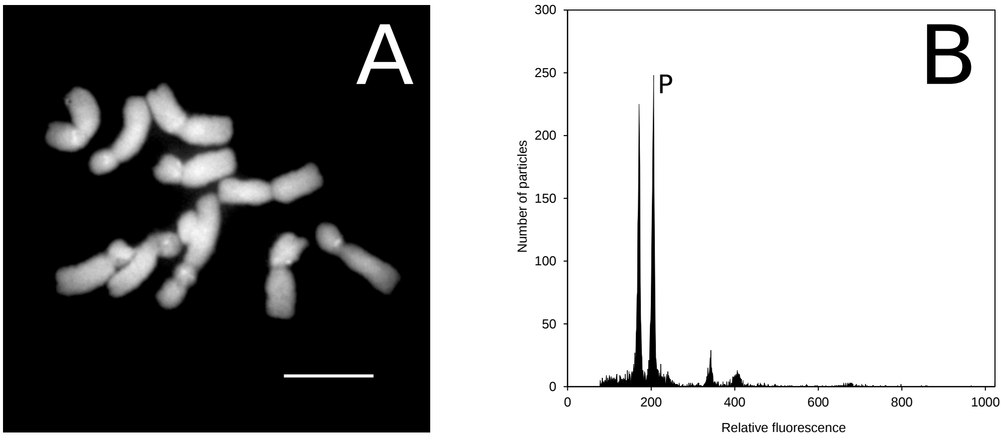

A+B. "Mediterranean diploid", population MK02, 2n = 10, 2C = 7.362 pg.

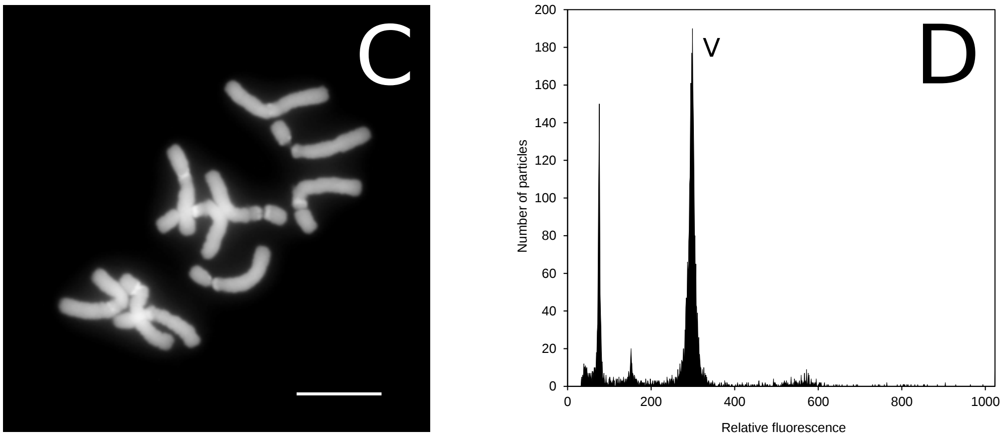

C+D. *Anthoxanthum aristatum* / *ovatum*, population PT07, 2n = 10, 2C = 7.027 pg.

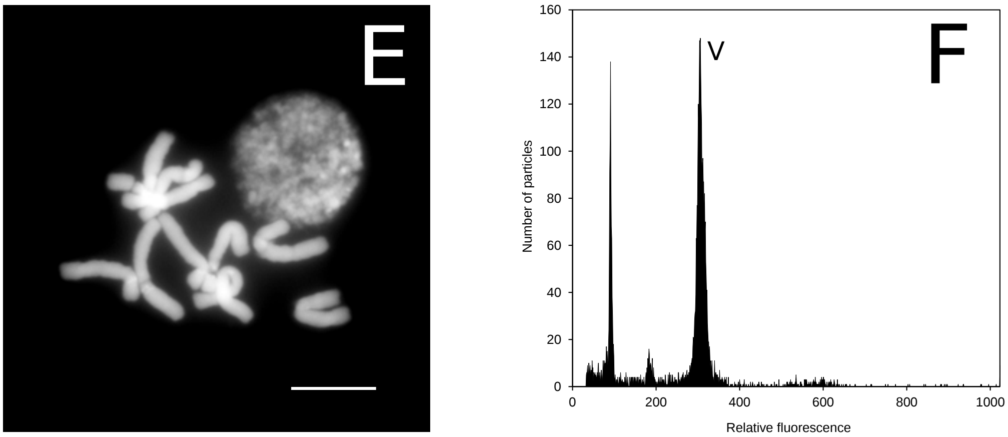

E+F. *Anthoxanthum aristatum* / *ovatum*, population FR12, 2n = 10, 2C = 8.299 pg.

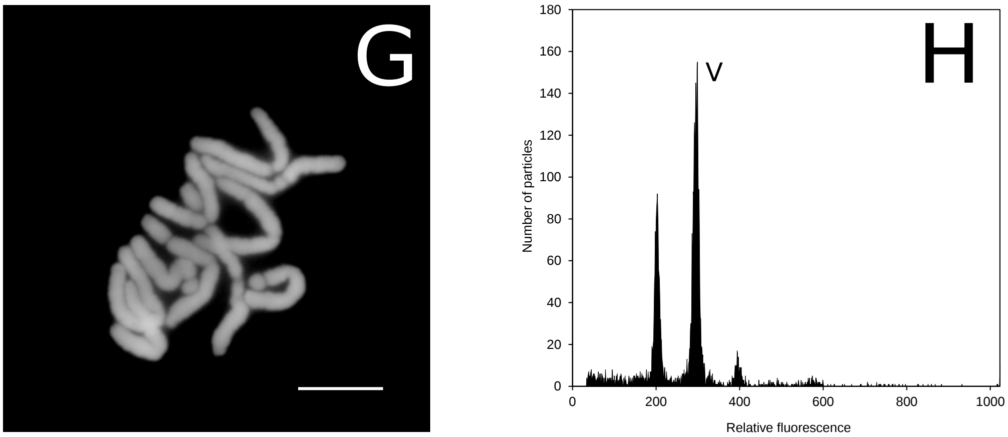

G+H. *Anthoxanthum gracile*, population GR09, 2n = 10, 2C = 18.633 pg.

Note: Micrograph's scale bar 10 µm; Letters in histograms determine standard plant (P - *Pisum sativum*, V - *Vicia faba*).
