# Supplementary figures and images for: Evolutionary and Taxonomic Implications of Variation in Nuclear Genome Size: Lesson from the Grass Genus Anthoxanthum (Poaceae)
Source: PLoS One. 2015 Jul 24;10(7):e0133748. doi: 10.1371/journal.pone.0133748 (PMC4514812; doi:10.1371/journal.pone.0133748)

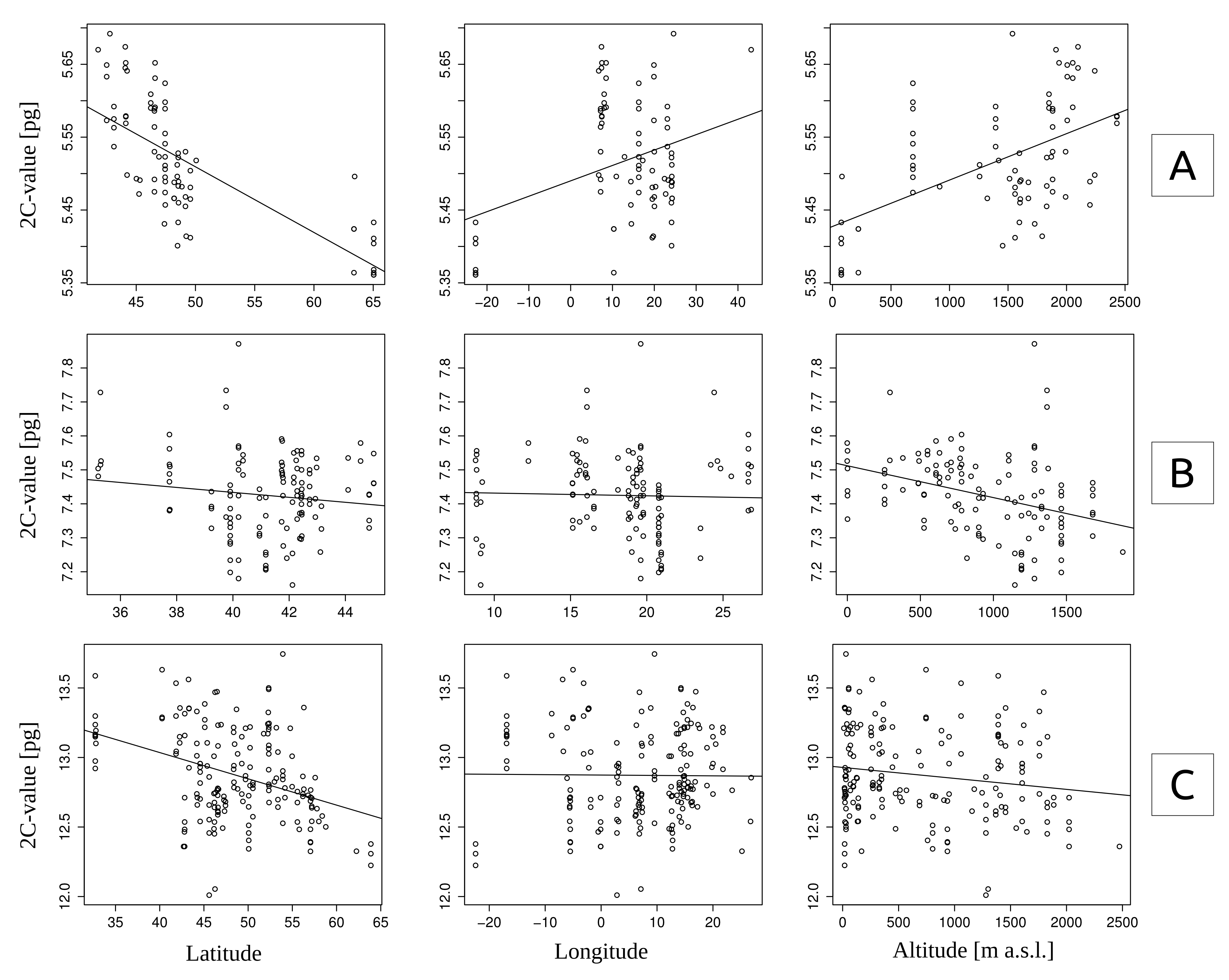

Supplement: S1 Fig — (A) 2x Anthoxanthum alpinum; (B) “Mediterranean diploid”; (C) 4x Anthoxanthum odoratum. (TIF) [file pone.0133748.s003.tif]
