# Supplementary material for: Evolutionary and Taxonomic Implications of Variation in Nuclear Genome Size: Lesson from the Grass Genus Anthoxanthum (Poaceae)
Source: PLoS One. 2015 Jul 24;10(7):e0133748. doi: 10.1371/journal.pone.0133748 (PMC4514812; doi:10.1371/journal.pone.0133748)
Supplement: S1 Table — For each population the information is provided about geography (population code, locality detail, coordinates in WGS-84 system and altitude), collector initials, number of analysed plants, mean holoploid genome size with standard deviation (in picograms of DNA) and intrapopulation variation (%). (PDF) [file pone.0133748.s004.pdf]

Table S1. List of analysed *Anthoxanthum* populations (sorted by ploidy level and holoploid genome size for each recognized taxonomic group). For each population the information is provided about geography (population code, locality detail, coordinates in WGS-84 system and altitude), collector initials, number of analysed plants, mean holoploid genome size with standard deviation (in picograms of DNA) and intrapopulation variation (%).

Taxon	State	Population code	Locality	Coordinates	Altitude [m a.s.l.]	Collector	N-FCM	GS ± s.d. [pg]	Variation [max/min; %]
***Anthoxanthum alpinum* 2x**									
	**Austria**								
		AT01	Gefrorener see	N47.4522; E14.3985	2200	JK	2	5.473 ± 0.023	0.6
		AT02	Hohe Tauren. Almhütte Laderalm	N46.9213; E12.8772	1870	HC	1	5.523	-
		*AT03	Burgenland. Redlschlag	N47.4367; E16.2883	687	PT, ZC	10	5.542 ± 0.049	2.7
		AT04	Steiermark. St. Johann am Tauern	N47.3831; E14.5209	1728	PV	1	5.431	-
	**Bulgaria**								
		BG01	Berkovitsa. Petrochan	N43.1184; E23.1268	1394	AK, FKr	4	5.567 ± 0.023	1
		BG02	Stara Planina. Trojanski prochod (Beklemeto)	N42.7798; E24.6063	1538	AK, FKr	1	5.692	-
	**Czech Republic**								
		*CZ01	Jeseníky. Velká Kotlina	N50.0600; E17.2371	1420	MS	1	5.518	-
	**France**								
		FR01	Provence-Alpes-Côte d'Azur. Lac D'Allos	N44.2291; E6.7112	2241	PT	2	5.57 ± 0.101	2.6
		FR02	Provence-Alpes-Côte d'Azur. Vallee des Merveilles	N44.0766; E7.3677	2098	PT	2	5.66 ± 0.021	0.5
		FR03	Provence-Alpes-Côte d'Azur. Vallon de la Valmasque	N44.1159; E7.4717	1936	PT	1	5.652	-
		FR04	Provence-Alpes-Côte d'Azur. Lac de L'Agnel	N44.1234; E7.4424	2430	PT	3	5.575 ± 0.006	0.2
	**Georgia**								
		GE01	Samckhe Javakheti. Borjomi Nature Reserve	N41.7862; E43.1603	1910	MS	1	24593	-
	**Iceland**								
		IS01	Halgefellssveit	N65.0426; W22.7279	75	JS, RS	7	5.386 ± 0.029	1.3
	**Montenegro**								
		ME01	Šcápica	N42.5075; E19.9093	2006	FR	3	5.618 ± 0.04	1.4
	**Norway**								
		NO01	Sør-Trøndelag. Røros. Granåsen	N63.3752; E10.3172	221	RE	3	5.404 ± 0.035	1.1
		NO02	Sør-Trøndelag. Røros. Stormyra	N63.4341; E10.9136	81	RE	1	5.496	-
	**Poland**								
		PL01	Babia Gora	N49.5738; E19.5330	1571	FK, MS	4	5.466 ± 0.039	1.7
	**Romania**								
		RO01	Lupeni	N45.3110; E23.3298	1610	JK	1	5.491	-
		RO02	Lacul Iovanu	N45.2382; E22.7213	1560	JK	1	5.472	-
		RO03	Prisacina	N45.0160; E22.4519	1513	JK	1	5.493	-
	**Slovakia**								
		SK01	Vysoké Tatry. Furkotská dolina	N49.1542; E20.0292	1830	PV	1	5.455	-
		SK02	Vysoké Tatry. Kriváň	N49.1581; E19.9937	1992	PV	2	5.499 ± 0.044	1.1
		SK03	Roháče. Zábrať	N49.2182; E19.7553	1792	PV	1	5.414	-
		SK04	Slovenský Raj. Stratená	N48.8541; E20.3077	916	JS	1	5.482	-
	**Switzerland**								
		CH01	Fribourg. La Gruyere. Vanil Noir	N46.5370; E7.1624	1881	PT, ZC	6	5.539 ± 0.048	2.1
		CH02	Ticino. Bellinzona. Chalt Herbrig	N46.5904; E8.4724	2053	PT	3	5.625 ± 0.031	1.1
		CH03	Valais. Simplon Kulm	N46.2280; E8.0132	1850	PT	3	5.599 ± 0.01	0.3

Taxon	State	Population code	Locality	Coordinates	Altitude [m a.s.l.]	Collector	N-FCM	GS ± s.d. [pg]	Variation [max/min; %]
***Anthoxanthum alpinum* 2x**									
	**Ukraine**								
		UA01	Svidovec. Bliznica	N48.2064; E24.2501	1674	PR	2	5.477 ± 0.016	0.4
		UA02	Svidovec. Kvasy	N48.1911; E24.2576	1323	PR	1	5.466	-
		UA03	Gorgany. Pogar	N48.5698; E24.0918	1602	PR	2	5.475 ± 0.021	0.5
		UA04	Gorgany. Sivulya	N48.5445; E24.1240	1714	PR	4	5.492 ± 0.044	1.7
		UA05	Gorgany. Taupisz	N48.4892; E24.1279	1454	PR	1	5.401	-
		UA06	Gorgany. Bystrica	N48.4637; E24.1844	1257	PR	2	5.504 ± 0.011	0.3
A. maderense									
	**Portugal**								
		PT01	Madeira. Pico do Arieiro	N32.7421; W16.9388	1602	PV, KK	6	6.967 ± 0.07	2.4
		PT02	Madeira. Rabacal	N32.7574; W17.1315	1181	PT, ZC	12	6.98 ± 0.074	3.9
		PT03	Madeira. Levada do Caldeirão Verde	N32.7819; W16.9285	901	PT, ZC	6	6.951 ± 0.068	2.8
		PT04	Madeira. Rabacal. Risco. 25 Fontes	N32.7614; W17.1287	1054	PT, ZC	2	6.873 ± 0.034	0.7
		PT05	Madeira. Pico Ruivo	N32.7483; W16.9387	1670	PT, ZC	25	6.929 ± 0.111	8.6
**"Mediterranean diploid"**									
	**Albania**								
		AL01	Qafa e Pejes	N42.4496; E19.7698	1680	FR	6	7.396 ± 0.058	2.1
		AL02	Llogara	N40.2005; E19.6018	1281	FR	9	7.469 ± 0.205	9.6
	**Bosnia and Herzegovina**								
		BA01	Kakanj	N44.0974; E18.1091	380	PT	2	7.488 ± 0.066	1.3
	**Bulgaria**								
		BG03	Pirin. Dolno Dragilište	N41.9170; E23.5213	819	AK, FKr	2	7.284 ± 0.062	1.2
	**Croatia**								
		HR01	Lika-Senj. Brinje	N45.0076; E15.1287	486	PT	3	7.49 ± 0.051	1.2
		HR02	Lika-Senj. Gorici	N44.8457; E15.1469	525	PT	4	7.384 ± 0.051	1.4
	**France**								
		FR05	Corse. Zonza	N41.7913; E9.2114	1037	PT	2	7.37 ± 0.133	2.6
		FR06	Corse. Manso	N42.4277; E8.8231	929	PT	3	7.381 ± 0.074	1.8
		FR07	Corse. Calenzana	N42.4405; E8.8431	602	PT	4	7.5 ± 0.072	2.1
		FR08	Corse. Evisa	N42.2504; E8.7755	291	PT	1	7.528	-
		FR09	Corse. Tattone	N42.1181; E9.1090	1147	PT	3	7.273 ± 0.123	3.4
	**Greece**								
		GR01	Crete. Ano Malaki	N35.2874; E24.4185	292	PT, JK	1	7.728	-
		GR02	Crete. Ammoudari	N35.2916; E24.1955	683	PT, JK	1	7.515	-
		GR03	Crete. Lassithi - Exo Potamoi	N35.2109; E25.5408	847	PT, JK	1	7.481	-
		GR04	Crete. Psiloritis - Nida	N35.2067; E24.8350	1375	PT, JK	1	7.504	-
		GR05	Crete. Arkadi	N35.3096; E24.6316	491	PT, JK	1	7.526	-
		GR06	Epirus. Vradeto	N39.9049; E20.7827	1465	TU, PV, KK	12	7.337 ± 0.08	3.6
		GR07	Samos. Marathokampos. by the dirty road to Mt. Kerkis	N37.7430; E26.6620	782	PT, ZC	6	7.503 ± 0.078	3.1
		GR08	Samos. Vourliotes. by the dirty road to Mt. Ampelos	N37.7570; E26.8445	878	PT, ZC	2	7.447 ± 0.09	1.7

Taxon	State	Population code	Locality	Coordinates	Altitude [m a.s.l.]	Collector	N-FCM	GS ± s.d. [pg]	Variation [max/min; %]
**Mediterranean diploid**									
	**Italy**								
		IT01	Apulia. Foggia. Valico del Lupo	N41.7797; E16.0931	689	PT	2	7.451 ± 0.037	0.7
		IT02	Apulia. Foggia. Monte Sant Angelo	N41.7557; E15.9881	607	PT	4	7.519 ± 0.046	1.3
		IT03	Apulia. Foggia. San Marco In Lamis	N41.7331; E15.6011	710	PT	4	7.49 ± 0.103	3.3
		IT04	Calabria. Lago Arvo	N39.2360; E16.5316	1330	PT	4	7.386 ± 0.044	1.5
		IT05	Calabria. Lungro	N39.7582; E16.0748	1367	PT	3	7.593 ± 0.203	5.1
		IT06	Campania. Piaggine	N40.3596; E15.4251	1105	PT	3	7.519 ± 0.03	0.8
		IT07	Emilia-Romagna. Casalborsetti	N44.5408; E12.2378	-1	PT	2	7.553 ± 0.037	0.7
	**Makedonia**								
		MK01	Pelagonia. Gorno Krushje	N41.1693; E20.9453	1192	TU, PV, KK	8	7.266 ± 0.081	3
		MK02	Pelagonia. Trpejca	N40.9447; E20.7898	900	TU, PV, KK	5	7.362 ± 0.064	1.9
	**Montenegro**								
		ME02	Budva	N42.2846; E18.8031	1	PT	5	7.455 ± 0.077	2.7
		ME03	Sutumore	N42.1657; E19.0994	631	PT	3	7.497 ± 0.047	1.2
		ME04	Velje Duboko	N42.7278; E19.3452	255	PT	5	7.45 ± 0.045	1.4
		ME05	Majstori	N42.3791; E18.8394	1242	PT	2	7.335 ± 0.052	1
		ME06	Durmitor. Žabljak	N43.1112; E19.0191	1886	JC	1	7.258	-
		ME07	Nikšić. Jasenovo Polje	N42.9320; E18.9366	1096	PT	2	7.388 ± 0.038	0.7
		ME08	Pljevlja. Kosanica	N43.1482; E19.3007	739	PT	2	7.36 ± 0.047	0.9
		ME09	Mojkovac. Donja Polja	N42.9771; E19.5384	772	PT	2	7.521 ± 0.019	0.4
Anthoxanthum aristatum / ovatum									
	**France**								
		FR10	Corse. Giuncheto	N41.5544; E8.8853	52	PT	16	8.045 ± 0.312	13.7
		FR11	Corse. Macinaggio	N42.9704; E9.4529	12	PT	37	7.604 ± 0.643	56.3
		FR12	Corse. Bonifacio	N41.4071; E9.2131	4	PT	12	8.299 ± 0.408	17
	**Portugal**								
		PT06	Fiais de Beira	N40.4253; W7.9371	153	PT, JK, JZ	4	7.099 ± 0.087	3
		PT07	Beira Litoral. Coimbra. Parrozelos	N40.2086; W7.8996	864	PS	4	7.027 ± 0.177	5.4
	**Spain**								
		ES01	Cuenca Alta del Lozoya. Madarcos	N41.0367; W3.5686	1067	PT, JK, JZ	3	7.128 ± 0.251	6.7
		ES02	Sierra de Grazalema. Ubrique	N36.6114; W5.4272	749	PT, JK, JZ	2	7.663 ± 0.188	3.5
		ES03	NP Los Alcornocales	N36.5598; W5.6011	449	PT, JK, JZ	5	7.529 ± 0.189	5.3
		ES04	Algeciras. NP Los Alcornocales	N36.1450; W5.5913	222	PT, JK, JZ	1	7.474	-
		ES05	Doňana. Matalascanas	N37.0479; W6.5693	25	PT, JK, JZ	2	7.544 ± 0.086	1.6
		ES06	Doňana. Mazagon	N37.1447; W6.8109	35	PT, JK, JZ	20	7.62 ± 0.396	20.1
		ES07	Doňana. Almonte	N37.2132; W6.4426	35	PT, JK, JZ	24	7.458 ± 0.294	15.7
		ES08	Monfragüe. Rio Tajo	N39.8282; W6.0504	450	PT, JK, JZ	4	7.265 ± 0.515	14.9
		ES09	Monfragüe. Rio Tietar	N39.8412; W5.9659	258	PT, JK, JZ	13	7.877 ± 0.88	37.9
Anthoxanthum gracile									
	**Greece**								
		GR09	Crete. Kournas	N35.3181; E24.3075	299	PT, JK	1	18.633	-
		GR10	Crete. Imbros gorge	N35.2446; E24.1669	741	PT, JK	4	18.315 ± 0.443	5.5

Taxon	State	Population code	Locality	Coordinates	Altitude [m a.s.l.]	Collector	N-FCM	GS ± s.d. [pg]	Variation [max/min; %]
Anthoxanthum alpinum 4x									
	**France**								
		*FR05	Auvergne. Mont Dore	N45.5963; E2.8343	1280	PT, ZC	1	11.069	-
		FR13	Auvergne. Puy Mary	N45.1127; E2.6728	1612	PT, ZC	4	11.081 ± 0.091	1.8
		FR14	Auvergne. Les Estables	N44.9117; E4.1870	1649	PT, ZC	3	10.981 ± 0.088	1.5
		FR15	Rhóne-Alpes. Charmant som	N45.3209; E5.7610	1771	PT, ZC	1	11.125	-
	**Switzerland**								
		CH04	Valais. Les Crossets	N46.1909; E6.8360	1828	PT, ZC	3	10.731 ± 0.214	4.1
		*CH05	Vaud. Morges. Mont Tendre	N46.5937; E6.3068	1637.2	PT, ZC	5	10.973 ± 0.063	1.4
		*CH06	Valais. Chatel	N46.2592; E6.8545	1797	PT, ZC	1	10.871	-
		CH07	Vaud. La Dōle	N46.4225; E6.1014	1597.5	PT, ZC	2	11.217 ± 0.18	2.3
Anthoxanthum odoratum									
	**Austria**								
		*AT03	Burgenland. Redlschlag	N47.4367; E16.2883	687	PT, ZC	2	12.669 ± 0.019	0.2
		AT05	Lower Austria. Ötzbach	N48.3889; E15.3030	700	PT, ZC	1	12.941	-
		AT06	Lower Austria. Dürnstein	N48.3976; E15.5211	327	PT, ZC	2	12.803 ± 0.027	0.3
		AT07	Salzburg. Uttendorf	N47.2568; E12.5824	830	TU	1	12.72	-
		AT08	Salzburg. Hochkogel	N47.3329; E12.6079	1156	TU	1	12.614	-
	**Croatia**								
		HR03	Karlovac. Janja Gora	N45.0968; E15.4345	364	PT	3	13.291 ± 0.086	1.3
	**Czech Republic**								
		*CZ01	Jeseníky. Velká Kotlina	N50.0600; E17.2371	1420	MS	1	12.645	-
		CZ02	Slavkovský les. Křížky	N50.0659; E12.7518	804	PT	4	12.509 ± 0.16	3.1
		CZ03	Slavkovský les. Dominova skalka	N50.0714; E12.7863	759	PT	1	12.404	-
		CZ04	České středohoří. Opárno	N50.5392; E13.9886	271	PT	4	12.743 ± 0.114	1.9
		CZ05	Havraníky	N48.8123; E15.9954	328	PT	2	12.9 ± 0.178	2
		CZ06	Chanovice	N49.4111; E13.7311	530	JS, RS	1	12.684	-
		CZ07	Central Bohemia. Vlašim	N49.7080; E14.8848	347	ZC	3	12.973 ± 0.205	2.9
	**Denmark**								
		DK01	Hovedstaden. Eremitageslottet	N55.7878; E12.5780	30	MP	4	12.586 ± 0.116	2
		DK02	Møns Klint, forest SE of autocamping	N54,9765; E12,5256	97	FK, EZ	2	12.900 ± 0.155	1.7
	**Estonia**								
		EE01	Pölva County. Kanepi	N57.9989; E26.7236	133	PK	1	20059	-
		EE02	Vöru County. Krabi	N57.5843; E26.8339	125	PK	1	12.855	-
	**Finland**								
		FI01	Petäjävesi	N62.2500; E25.1846	169	HC	1	12.326	-
	**France**								
		*FR05	Auvergne. Mont Dore	N45.5963; E2.8343	1280	PT, ZC	5	12.508 ± 0.316	7.1
		FR16	Auvergne	N45.3726; E2.8078	1413	PT, ZC	2	13.022 ± 0.117	1.3
		FR17	Auvergne. Les Salces	N44.5720; E3.1159	1604	PT, ZC	7	12.828 ± 0.156	3.3
		FR18	Rhóne-Alpes. Haute-Savoie. Le Corbier	N46.2835; E6.6453	1246	PT, ZC	1	12.746	-
		FR19	Provence-Alpes-Côte d'Azur. Allos	N44.2531; E6.6831	1886	PT	2	12.686 ± 0.037	0.4
		FR20	Bretagne. Plévenon	N48.6857; W2.3187	56	PT	1	13.346	-
		FR21	Pyrenees. Vallee du Lutour	N42.8209; W0.0899	2023	JK	4	12.523 ± 0.146	2.8
		FR22	Hautes-Pyrénées. Port de Boucharo	N42.6995; W0.0607	2473	JK	1	12.36	-
		FR23	Corse. Nueve	N42.2908; E8.8747	1456	PT	1	13.356	-
		FR24	Corse. Cristinacce	N42.2557; E8.9185	1709.5	PT	2	13.127 ± 0.031	0.3

Taxon	State	Population code	Locality	Coordinates	Altitude [m a.s.l.]	Collector	N-FCM	GS ± s.d. [pg]	Variation [max/min; %]
Anthoxanthum odoratum									
	**Germany**								
		DE01	Brandenburg. Zossen	N52.2306; E13.4886	38	ZC, TC	2	13.206 ± 0.049	0.5
		DE02	Brandenburg. Kersdorf	N52.3250; E14.2692	54	ZC, TC	5	13.233 ± 0.26	4.6
		DE03	Brandenburg. Drehna	N51.7627; E13.8009	84	EZ	1	13.171	-
		DE04	Itzehoe, Kochschke Sandgrube	N53.9239; E9.5494	30	FK, EZ	5	12.977 ± 0.455	9.7
	**Great Britain**								
		GB01	Scotland. Luine Bheinn	N57.0504; W5.5141	936	HC	5	12.455 ± 0.141	2.9
		GB02	Scotland. Glenelg. Belvraid	N57.1973; W5.6111	140	HC	4	12.634 ± 0.069	1.3
		GB03	Scotland. Knoydart. Barrisdale	N57.0730; W5.5126	512	HC	2	12.74 ± 0.037	0.4
		GB04	England. Peak District NP	N53.3707; W1.8650	352	HC	3	12.793 ± 0.215	3.1
	**Hungary**								
		HU01	Veszprém. Sümeg	N46.9376; E17.3292	160	JC, ZD	1	13.236	-
		HU02	Nagykanizsa. Homokkomárom	N46.4840; E16.9262	150	JC, ZD	1	13.473	-
		HU03	Aggtelek	N48.4723; E20.4914	329	AK, FKr	1	13.096	-
	**Iceland**								
		IS02	Southern Peninsula. Blue Lagoon	N63.8815; W22.4454	18	JS, RS	3	12.303 ± 0.077	1.3
	**Ireland**								
		IE01	Leinster. Wicklow. Bray	N53.1912; W6.0874	129	HC	2	13.033 ± 0.257	2.8
	**Italy**								
		IT08	Piedmont. Cuneo. Valle di Valasco	N44.2003; E7.2370	1758	PT	4	13.046 ± 0.245	4.7
		IT09	Veneto. Belluno	N46.1383; E12.1685	473	PT	3	12.746 ± 0.261	4.2
	**Norway**								
		NO03	Rogaland. Lake Mjåvatnet	N58.3810; E6.1121	61	JS, RS	1	12.579	-
	**Poland**								
		PL02	Lesser Poland. Tatra County. Zakopane	N49.2844; E20.0045	951	PT, ZC	3	12.947 ± 0.206	3.2
		PL03	Lesser Poland. Krakòw County. Skała	N50.2224; E19.8128	445	PT, ZC	2	13.075 ± 0.204	2.2
		PL04	Silesian. Katowice-Panewniki	N50.2174; E18.9319	267	PT, ZC	2	12.934 ± 0.192	2.1
		PL05	Lubuskie w.. Rzepin	N52.3383; E14.8603	69	ZC, TC	5	12.98 ± 0.192	3.6
		PL06	Wielkoposkie w.. Bolewicko	N52.3644; E16.0903	94	ZC, TC	3	12.905 ± 0.3	4.5
		PL07	Lubuskie w.. Cybinka	N52.2798; E14.7004	53	EZ	1	13.324	-
		PL08	Kolobrzeg, parking place E of Grzybovo willage	N53.1630; E15,4517	17.1	FK, EZ	2	12.746 ± 0.049	0.6
		PL09	Łeba, pine forest E of the village	N54.7666; E17.5947	11	FK, EZ	1	13.210	-
		PL10	Chodziez, along the side roads close to the road Chodziedz -	N52.9784; E16.8187	101	FK, EZ	1	12.825	-
	**Portugal**								
		*PT08	Bragança. Rabal	N41.8659; W6.7525	679	MPP	1	13.044	-
		PT09	Madeira. Col du Poiso	N32.7125; W16.8854	1390	PV, KK	8	13.186 ± 0.205	5.2
		PT10	Madeira. Queimadas	N32.7844; W16.9052	883	PT, ZC	2	13.147 ± 0.065	0.7
	**Romania**								
		RO04	Runc. Cheile Pociovalistei	N46.4972; E23.4331	570	JK	1	12.764	-
	**Slovakia**								
		SK05	Tarbucka	N48.3628; E21.7896	254	JS	3	13.104 ± 0.165	2.3

Taxon	State	Population code	Locality	Coordinates	Altitude [m a.s.l.]	Collector	N-FCM	GS ± s.d. [pg]	Variation [max/min; %]
Anthoxanthum odoratum									
	**Spain**								
		ES10	Sierra de Gredos. Mombeltrán	N40.2671; W5.0257	745	PT, JK, JZ	3	13.4 ± 0.2	2.6
		ES11	Caňon del Rio Lobos. Hontoria de Pinar	N41.8446; W3.1256	1058	PT, JK, JZ	3	13.286 ± 0.254	3.9
		ES12	Basque. Getaria	N43.3097; W2.2018	22	PT, JK, JZ	3	13.21 ± 0.246	3.3
		ES13	Galicia. Noia. Nimo	N42.8339; W8.8328	256	MPP	1	13.158	-
		ES14	Galicia. Fraga de Nimo. Noia	N42.8328; W8.8314	291	MPP	1	13.315	-
		*ES15	Asturias. Pesoz	N43.2569; W6.8769	263	MPP	1	13.561	-
		ES16	Aragon. Candanchú	N42.8009; W0.5182	1646	JK	1	12.465	-
	**Sweden**								
		SE01	Öland, alvar in Mörbylilla	N56.3037; E16.4194	18	FK, EZ	2	13.066 ± 0.415	4.6
		SE02	Mörrum, railway E of Kråketorp station	N56.1907; E14.7171	23	FK, EZ	3	12.774 ± 0.079	1.1
		SE03	Simirshamn, coastal sands on the N border of the town	N55.5717; E14.3353	21	FK, EZ	3	12.656 ± 0.111	1.7
	**Switzerland**								
		CH08	Fribourg. La Gruyere. Gros Mont	N46.5540; E7.1994	1407	PT, ZC	1	12.728	-
		CH09	Neuchâtel. Métairie de Dombresson. Chasseral	N47.1220; E7.0353	1551	PT, ZC	1	12.492	-
		CH10	Valais. Les Crossets	N46.1909; E6.8360	1828	PT, ZC	4	12.576 ± 0.102	1.8
		*CH06	Valais. Chatel	N46.2592; E6.8545	1797	PT, ZC	1	13.469	-
		CH11	Bern. Obersimmental-Saanen. Jaunpass	N46.5926; E7.3487	1455	PT, ZC	3	12.777 ± 0.266	3.8
		CH12	Bern. Berner Jura. Forêt de Béroie	N47.2669; E7.1760	905	PT, ZC	1	12.693	-
		*CH05	Vaud. Morges. Mont Tendre	N46.5937; E6.3068	1618	PT, ZC	1	13.232	-
		CH13	Vaud. Jura-Nord vaudois. Le Brassus	N46.5664; E6.2342	1368	PT, ZC	3	12.66 ± 0.103	1.5
		CH14	Vaud. Aigle. Avançon	N46.2526; E7.1119	1300	PT, ZC	1	12.054	-
		CH15	Vaud. Bois de Ban. Fauchy	N46.6494; E6.3709	1176	PT, ZC	1	12.772	-
Anthoxanthum amarum									
	**Portugal**								
		PT11	Sao Joane	N40.7960; W8.2563	150	PT, JK, JZ	1	47.479	-
		PT12	Cepelos	N41.2514; W8.0937	163	PT, JK, JZ	2	44.939 ± 0.47	1.5
		PT13	Beira Litoral. Coimbra. Penacova. Agrêlo	N40.2833; W8.3500	258	PS	4	46.088 ± 0.651	3.1
		*PT08	Bragança. Rabal	N41.8659; W6.7525	679	MPP	1	46.574	-
		PT14	Cabeço da Neve. Caramulo	N40.5535; W8.1794	712	MPP	1	46.716	-
		PT15	Braganca. Road from Alimonte to Vila Boa de Ousilhao	N41.8025; W7.9369	879	MPP	1	44.846	-
		PT16	Sao Joao da Serra	N40.7803; W8.2403	373	MPP	1	43.848	-
		PT17	Almeida. Castelo Bom	N40.6200; W6.8956	696	MPP	1	42.185	-
		PT18	Caramulo. Caramulinho	N40.5478; W8.2017	961	MPP	1	46.202	-
		PT19	Bragança. road from Carregosa to Cova da Lua	N41.8800; W6.8128	825	MPP	1	39.513	-
		PT20	Extremo. near Monçao	N41.9647; W8.4736	406	MPP	1	44.713	-
	**Spain**								
		ES17	Galicia. Faìlde. Santa Comba	N43.0372; W8.8072	377	MPP	1	43.391	-
		ES18	Galicia. Pena do Coiro. Bueu	N42.2874; W8.7618	270	MPP	2	42.691 ± 0.226	0.8
		ES19	Galicia. Pambre. Palas de Rei. Lugo	N42.8561; W7.9506	467	MPP	2	44.447 ± 1.241	4
		ES20	Galicia. Zas	N43.1025; W8.9167	204	MPP	1	46.202	-
		*ES15	Asturias. Pesoz	N43.2569; W6.8769	263	MPP	2	42.718 ± 1.504	5.1
		ES21	Asturia. Road from taramundi to Oscos	N43.3167; W7.0500	869	MPP	1	45.351	-
		ES22	Asturia. La Coba. Grandas de Salime	N43.4259; W7.0346	345	MPP	1	41.547	-
		ES23	Cedeira. San Andrès de Teixido	N43.7086; W7.9825	159	MPP	1	49.74	-
		ES24	A Cabana. Belelle	N43.4650; W8.1072	287	MPP	1	43.542	-
		ES25	Ferrol. Valòn	N43.4942; W8.2664	105	MPP	1	47.08	-

* - mixed populations

Collectors: AK - Anna Krahulcová, EZ - Eliška Záveská, FK - Filip Kolář, FKr - Frantiček Krahulec, FR - Frederick Rooks, HC - Hana Chudáčková, JC - Jindřich Chrtek, JK - Jana Krejčíková, JS - Jan Suda, JZ - Jaroslav Zahradníček, KK - Karol Krak, MP - Martina Pičmanová, MPP - Manuel Pimentel Pereira, MS - Milan Štech, PK - Petr Kohout, PR - Pavla Růžičková, PS - Paulo Silveira, PT - Pavel Trávníček, PV - Petr Vít, RE - Reidar Elven, RS - Radka Sudová, TC - Tomáš Chum, TU - Tomáš Urfus, ZC - Zuzana Chumová, ZD - Zuzana Dočkalová.
