# Supplementary material for: Evolutionary and Taxonomic Implications of Variation in Nuclear Genome Size: Lesson from the Grass Genus Anthoxanthum (Poaceae)
Source: PLoS One. 2015 Jul 24;10(7):e0133748. doi: 10.1371/journal.pone.0133748 (PMC4514812; doi:10.1371/journal.pone.0133748)
Supplement: S2 Table — (PDF) [file pone.0133748.s005.pdf]

Table S2. List of published chromosome numbers for different species of the genus *Anthoxanthum*. (Ordered by taxon name, country of plant origin and ploidy level).

Taxon	Country	2n	References
A. alpinum **Löve & Löve**	Armenia	10	[77]
	Austria	10	[78-84]
	Bosnia-Herzegovina	10	[83]
	Bulgaria	10	[85-86]
	Croatia	10	[83]
	Czech Republic	10	[87-88]
	France	10	[83,89-94]
	Germany	10	[91,93-95]
	Great Britain	10	[96-99]
	Greece	10	[100]
	Greenland	10	[101-102]
	China	10	[103]
	Iceland	10	[104]
	Italy	10	[83,93-94]
	Japan	10	[103]
	North Korea	10	[103]
	Norway	10	[82,104-112]
	Poland	10; 10+1-2B	[107-110]
	Russia	10	[82,101,111-119]
	Slovakia	10	[119]
	Sweeden	10	[82,104,106]
	Switzerland	10; 9+2B; 15	[78,81-82,93-94,104,106,120-126]
	Turkey	10	[93]
	Ukraine	10	[127]
	France	20	[83,93-94,128]
	Switzerland	20	[93,123]

Taxon	Country	2n	References
**Diploid *A. odoratum* L. ("Mediterranean diploid")**	Algerie	10	[93]
	Bosnia-Herzegovina	10	[83]
	Croatia	10	[83,93-94]
	France	10	[89,94]
	Greece	10; 10+1B	[93,123,129]
	Italy	10	[83,93-94]
	Montenegro	10	[83]
	Serbia	10	[83]
	Slovenia	10	[93]
	Turkey	10	[93]
	Belgium	16	[130]
***A. odoratum* L.**	Austria	20	[78-79,81-82,84]
	Canada	20	[131-132]
	Czech Republic	20	[87,133]
	Danmark	20	[78,101,106,134]
	Finland	20	[135]
	France	20	[83,93-94,136]
	Germany	20	[78,93,95,137-138]
	Great Britain	20	[96-99,120,122,139-143]
	Greenland	20	[101]
	Hungary	20	[93,144]
	Iceland	20	[90,104,145]
	Ireland	20	[123]
	Italy	20	[93]
	Japan	20	[146-147]
	Marocco	20	[148-149]
	Netherlands	20	[150]
	Norway	20	[82,104,106,151-152]
	Poland	20	[107-109]
	Portugal	20	[83,93,153-155]

Taxon	Country	2n	References
	Romania	20	[83]
	Russia	20	[101,111,118,136,156-162]
	Slovakia	20	[163-165]
	Slovenia	20	[83,93,166]
	Spain	20	[78,93,149]
	Sweeden	20	[82,106,167-168]
	Switzerland	20; 21	[78,81-82,93,121,126]
	USA	20	[169]
	India	20+2B	[170]
***A. amarum* Brot.**	Portugal	80; 86; 88; 90	[104,122,129,153]
***A. maderense* Teppner**	Madeira	10	[171]
***A. ovatum* Lag.**	Italy	10	[97,122-123,142]
	Morocco	10	[104]
	Spain	10	[172]
***A. aristatum* Boiss.**	Maroco	10	[148]
	Portugal	10; 10+1-4B	[104,153,173]
	Spain	10; 10+1-2B	[172]
***A. gracile* Bivona**	Italy	10	[174]

REFERENCES

77. Pogosyan AI, Narinyan SG & Voskanyan VE. Data on the karyogeographical study of the flora of the Aragats massif. Biol Zhurn Armenii.1971;24: 37-43.

78. Böcher TW. Experimental and cytological studies on Plant species VI. *Dactylis glomerata* and *Anthoxanthum odoratum*. Bot Tidsskr. 1961;56: 314-335.

79. Polatschek A. Cytotaxonomische Beiträge zur Flora der Ostalpenländer. II. Oesterr Bot Z. 1966;113: 1-46.

80. Teppner H. *Anthoxanthum alpinum* und seine Verbreitung in der Steiermark. Phyton (Horn). 1969;13: 305-312.

81. Hedberg I. Cytotaxonomic studies on *Anthoxanthum odoratum* L. s. lat. III, Investigations of Swiss and Austrian population samples. Svensk Bot Tidskr. 1969;63: 233-256.

82. Hedberg I. Cytotaxonomic studies on *Anthoxanthum odoratum* L. s. lat. IV. Karyotypes, meiosis and the origin of tetraploid *A. odoratum*. Hereditas. 1970;64: 153-176.
83. Teppner H. Karyotypen europäischer, perennierenden Sippen der Gramineen-Gattung *Anthoxanthum*. Oesterr Bot Z. 1970;118: 280-292.
84. Hedberg I. Morphological, cytotaxonomic and evolutionary studies in *Anthoxanthum odoratum* L. s. lat. – a critical review. Sommerfeltia. 1990;11: 97-107.
85. Pundeva RS. Karyological study on two varieties of *Anthoxanthum odoratum* L. var. *odoratum* and var. *montanum* Aschers et Graebn. Dokl Bolg Akad Nauk. 1974;27: 395-398.
86. Pundeva R. Morfologichno i kariologichno prouchvane na rod *Anthoxanthum* L. v Bulgariya. [Morphological and karyological study on the *Anthoxanthum* L. genus in Bulgaria]. Rasteniev Nauki. 1975;12: 123-130.
87. Mayová M. Příspěvek k řešení problematiky *Anthoxanthum odoratum* L. s. lat. se zvláštním zřetelem k *Anthoxanthum alpinum* A. & D. Löve. M. Sc. Thesis. Charles University in Prague, Prague. 1982.
88. Krahulcová A. Selected chromosome counts of the Czechoslovak flora II. Folia Geobot Phytotax. 1990;25: 381-388.
89. Contandripoulos J. Caryologie et localization des espéce végétales endémique de la Corse. Bull Soc Bot France. 1957;104: 53-55.
90. Löve Á & Löve D. The diploid perennial *Anthoxanthum*. Sci Iceland. 1968; 26-30.
91. Derchs G. Über einigen Chromosomenzählungen an mitteleuropäischen Blütenpflanzen. Philippia. 1974;2: 75-85.
92. Löve Á. IOPB chromosome number reports. XLIV. Taxon. 1974;23: 373-380.
93. Felber F. Contribution à l'étude phytogéographique, biosystématique et experimentale du complexe polyploïd *Anthoxanthum odoratum* L. s. lat. [Contribution to the study of phytogeography, biosystematics and experiments in the polyploid complex of *Anthoxanthum odoratum* L. s. lat.]. Thèse à l'UNINe [PhD thesis], Neuchâtel. 1987.
94. Felber F. Distribution des cytodémes d'*Anthoxanthum odoratum* L. s. lat. en France et dans les régions limitrophes. Bull Soc Bot France. 1988;135: 281-293.
95. Bogenrieder A & von Stietencron A. Morphologische und cytologische Untersuchungen an *Anthoxanthum alpinum* A. & D. Löve und *Anthoxanthum odoratum* L. vom Feldberg im Schwarzwald. Carolinea. 1985;42: 51-56.
96. Jones K. Interchange heterozygosity in *Anthoxanthum*. Heredity. 1961;16: 523.

97. Borrill M. The experimental taxonomy of *Anthoxanthum* species. Proc Linn Soc London. 1962;173: 106-109.
98. Jones BMG. *Anthoxanthum alpinum* A. and D. Löve, new to the British Isles. Nature. 1963;198: 610.
99. Jones BGM. & Melderis A. *Anthoxanthum odoratum* L. and *A. alpinum* Á. & D. Löve. Bot Soc Brit Isles Proc. 1964;5: 375-377.
100. Strid A & Franzen R. In: Löve Á, editor. IOPB chromosome number reports. LXIII. Taxon. 1981;30: 829-842.
101. Böcher TW & Larsen K. Chromosome numbers of some arctic or boreal flowering plants. Meddelelser om Grönland. 1950;147: 1-32.
102. Jörgensen CA, Sörensen TH & Westwrgaard M. The flowering plants of Greenland. A taxonomical and cytological survey. Biol Skr. 1958;9: 1-172.
103. Tateoka T. Notes on *Anthoxanthum nipponicum* Honda. J Jpn Bot. 1966;41: 85-88.
104. Östergren G. Chromosome numbers of *Anthoxanthum*. Hereditas. 1942;28: 242-243.
105. Knaben G. Chromosome numbers of Scandinavian Arctic-alpine plant species. I. Blyttia. 1950;8: 129-155.
106. Hedberg I. Cytotaxonomic studies on *Anthoxanthum odoratum* L. s. lat. II. Investigations of some Swedish and a few Swiss population samples. Symb Bot Upsal. 1967;8: 1-88.
107. Skalińska M, Banach-Pogan E, Weisło H et al. Further studies in chromosome numbers of Polish angiosperms. Acta Soc Bot Poloniae. 1957;26: 215-246.
108. Rozmus M. Cytological investigations on *Anthoxanthum alpinum* L. et L., a new species of the flora of Poland. Acta Biol Cracov, Ser Bot. 1958;1: 171-184.
109. Rozmus M. The taxonomical rank of *Anthoxanthum alpinum* L. & L. in the light of the anatomical studies. Acta Biol Cracov, Ser Bot. 1961;3: 81-90.
110. Rozmus M. Badania cytogenetyczne nad biotypami *Anthoxanthum alpinum* L. et L. z dodatkowymi chromosomami. [Cytogenetical studies in biotypes of *Anthoxanthum alpinum* with accessory chromosomes]. Acta Biol Cracov, Ser Bot. 1963;6: 115-141.
111. Sokolovskaya AP. Velichina pyltsevich zeren i chisla khromosom u nekotorykh arkticheskikh vidov zlakov [Size of pollen grains and chromosome numbers of some arctic species of grasses]. Bot Zhurn. 1955;40: 850–853.
112. Sokolovskaya AP & Strelkova OS. Geograficheskoe rasprostranenie poliploidnykh vidov rasteniï v evroazitskoï arktike [Geographical distribution polyploid plant species in the Euroasian Arctic]. Bot Zhurn. 1960;45: 370-381.

113. Belaeva VA & Siplivinsky VN. In: Goldblatt P, editor. Index to plant chromosome numbers 1982-1983. Missouri Botanical Garden. 1985.
114. Krogulevich RE. In: Goldblatt P, editor. Index to plant chromosome numbers 1982-1983. Missouri Botanical Garden. 1985.
115. Magulaev AV. In: Goldblatt P, editor. Index to plant chromosome numbers 1982-1983. Missouri Botanical Garden. 1985.
116. Gvinianidze ZI & Avaznelli AA. In: Goldblatt P, editor. Index to plant chromosome numbers 1982-1983. Missouri Botanical Garden. 1985.
117. Probatova NS & Sokolovskaya AP. In: Goldblatt P, editor. Index to plant chromosome numbers 1982-1983. Missouri Botanical Garden. 1985.
118. Marhold K. IAPT/IOPB chromosome data 13. Taxon. 2012;61: 889-902.
119. Hindáková M. In Májovský J et al., editors. Index of chromosome numbers of Slovakian flora. (Part 4). Acta Fac Rerum Nat Univ Comenianae, Bot. 1974;22: 1-20.
120. Tutin TG. A note on species pairs in the Gramineae. Watsonia. 1950;1: 224-227.
121. Favarger C. Contribution de la biosystématique à l'étude des flores alpine et jurassienne. Rev Cytol Biol Veg. 1962;25: 397-410.
122. Borrill M. Experimental studies of evolution in *Anthoxanthum* (Gramineae). Genetica. 1963;34: 183-210.
123. Jones K. Chromosomes and the nature and origin of *Anthoxanthum odoratum* L. Chromosoma. 1964;15: 248-274.
124. Hedberg I. Pubescence – a spurious taxonomic character in *Anthoxanthum odoratum* L. s. lat. Svensk Bot Tidskr. 1964;58: 237-241.
125. Scholte G. In: Löve Á., editor. IOPB chromosome number reports. LVI. Taxon. 1977;26: 257-274.
126. Felber F & Girard M. Etude écologique et génétique d'une zone de contact entre deux cytodemes *d'Anthoxanthum odoratum* L. s. lat. à Chasseral (Berne, Suisse). Resultát préliminaires. In.: Actes du Colloque Biologie des populations. Colloque national du C.N.R.S. Lyon, Université Claude Bernard. 1986; 330-331.
127. Pashuk KT. Cytological investigation of the *Anthoxanthum* genus in the Carpathians. Ukrayins'k Bot Zhurn. 1970;27: 429.
128. Guinochet M. Recherches de taxonomie expérimentale sur la flore des Alpes et la région méditerranéenne occidentale. I. Notes caryologiques sur quelques Graminées. Rev Cytol Cytophysiol Veg. 1943;6: 209-220.

129. Teppner H. Poaceae in the greenhouses of the Botanic Garden of the Institute of Botany in Graz (Austria, Europe). Fritschiana. 2002;31: 1-42.
130. Marchal E. Recherches sur les variations numériques des chromosomes dans la série végétale. Mémoires de la Classe des science: Académie royale de Belgique; 1920.
131. Bowden WM. Chromosome numbers and taxonomic notes on northern grassess. III. Twenty-five genera. Canad J Bot. 1960;38: 541-557.
132. Taylor RL & Mulligan GA. Flora of the Queen Charlotte Islands, Vol. 2, Cytological aspects of the vascular plants. Ottawa: Queen's Printer; 1968.
133. Kirschner J, Štěpánek J & Štěpánková J. In: Löve Á., editor. IOPB chromosome number reports. LXXVI. Taxon. 1982; 31: 574-598.
134. Böcher TW. Zur Zytologie eniger arktischen und borealen Blütenpflanzen. Svensk Bot Tidskr. 1938;32: 346-361.
135. Sorsa V. Chromosomenzahlen Finnischer Kormophyten. I. Ann Acad Sci Fenn, Ser. A, IV, Biol. 1962;58: 1-14.
136. Küpfer P. Nouvelles prospections caryologiques dans la flore orophile des Pyrénées et de la Sierra Nevada. Bull Soc Neuchâteloise Sci Nat. 1968;91: 87-104.
137. Kattermann G. Über die Bildung polyvalenter Chromosomenverbände bei einigen Gramineen. Planta. 1931;12: 732-774.
138. Rohweder H. Versuch zur Erfassung der mengenmässigen Bedeckung des Darss und Zingst mit polyploiden Pflanzen. Ein Betraig zur Bedeutung der Polyploidie bei der Eroberung neuer Lebensräume. Planta. 1937;27: 501-549.
139. Parthasarathy N. Cytogenetical studies in Oryzeae and Phalarideae. III. Cytological studies in Phalarideae. Ann Bot. 1939;3: 43-77.
140. Hubbard CE. Grasses. A guide to their structure, identification, uses, and distribution in the British Isles. Hardmondworth, Middlesex: Penguins book Ltd.; 1954.
141. Hedberg O. Cyto-taxonomic studies in Scottish mountain plants, notably *Deschampsia caespitosa* (L.) P. B., s. lat. Svensk Bot Tidskr. 1958;52: 37-46.
142. Jones K. Chromosomal status, gene exchange and evolution in *Dactylis*. I. The chromosomal analysis of diploid, tetraploid and hexaploid species and hybrids. Genetica. 1962;32: 272-295.
143. Richards AJ. In: Löve Á, editor. IOPB chromosome number reports. XXXVI. Taxon. 1972;21: 333-346.

144. Pólya L. Magyarországi növényfajok kromoszómaszámai. II [Chromosome numbers of Hungarian plants. II]. Ann Biol Univ Debrecen. 1950;1: 46-56.
145. Löve Á & Löve D. Cytotaxonomical conspectus of the Icelandic flora. Acta Horti Gothob. 1956;20: 65-291.
146. Tateoka T. Karyotaxonomic studies in Poaceae. II. Annu Rep Natl Inst Genet Jpn. 1954;5: 68-69.
147. Tateoka T. Karyotaxonomic studies in Poaceae. III. Annu Rep Natl Inst Genet Jpn. 1956;6: 73-74.
148. Miège J. Contribution à l'étude des Phalaridées. Bull Soc Hist Nat Afrique N. 1939;30: 223-245.
149. Valdès B. Numeros cromosomicos de algunas plantas españolas. I. Lagascalia. 1973;3: 211-217.
150. Gadella TWJ & Kliphuis E. Chromosome numbers of flowering plants in the Netherlands. Acta Bot Neerl. 1963;12: 195-230.
151. Laane MM. Kromosomundersökelser hos noen norske planter. Blyttia. 1965;23: 169-189.
152. Aaberge B. In: Knaben G, editor. Om kromosomvariasjon og rasedannelse i den norske flora. Blyttia. 1966;24: 65-79.
153. Fernandes A & Queirós M. Contribution à la connaissance cytotaxonomique des spérmatophyta du Portugal. IV. Leguminosae. Bol Soc Brot. 1969;45: 177-226.
154. Quierós M. Contribuição para o conhecimento citotaxonómico das spermatophyta de Portugal. I. Gramineae, Supl. 1. Bol Soc Brot. 1973;47: 77-103.
155. Quierós M. Contribuição para o conhecimento citotaxonómico das spermatophyta de Portugal. I. Gramineae, Supl. 2. Bol Soc Brot. 1974;48: 81-98.
156. Avdulov NP. Systematicheskaya karyologiya semeestva Gramineae [Systematic karyology of the Gramineae family]. Dnievnik vsesojuznogo Sezda Botanikov Leningrade [Diary of all-union botanical congress in Leningrad]. 1928: 65-67.
157. Avdulov NP. Karyo-systematische Untersuchungen der Familie Gramineen. Bull App Bot. 1931;43: 1-438.
158. Sokolovskaya AP & Strelkova OS. Geograficheskoe raspredelenie poliploidov. II. Issledovanie flory Altaya [Geographical distribution of polyploids. II. Investigation of the Altaï flora]. Uchen Zap Ped Inst Im Gertsena. 1948;66: 179-193.

159. Sokolovskaya AP & Strelkova OS. Geograficheskoe raspredelenie poliploidov. III. Issledovanie flory alpiiskoi oblasti tsentralnogo Kavkaznogo khrbeta [Geographical distribution of polyploids. III. Observation of alpine flora of the Central Caucasus mountain range]. Uchen Zap Ped Inst Im.Gertsena. 1948;66: 195-216.
160. Krogulevich RE. Rol poliploidii v genezise vysokogornoy flory Stanogovo Nagorya [The role of polyploidy in evolution of alpine flora of the Stanogovo Nagorye]. In.: Ekologiya flory Zabaykalya [Ecology of the flora of the Transbaikal territory]. Irkutsk: Akademiia Nauk SSSR, 1971; 115-214.
161. Sokolovskaya AP. Kariologicheskoe issledovanie flory Basseina R. Usy (Komi ASSR) [Karyological investigation of the flora of the Usa river basin (Komi ASSR)]. Vestn Leningradsk Univ, Ser Biol. 1970;2: 106-114.
162. Sokolovskaya AP. Kariologicheskaya kharakteristika predstavisteley flory leningradovskoy oblasti [Karyological characterization of some flora members of Leningrad territory]. Vestn Leningradsk Univ. 1972;21: 56-63.
163. Hadač E & Hašková V. Taxonomické poznámky o tatranských rostlinách ve vztahu k jejich cytologii. Biológia. 1956;11: 717-723.
164. Hindáková M. In: Májovský J et al., editors. Index of chromosome numbers of Slovakian flora (Part 1). Acta Fac Rer Nat Univ Com Bot. 1970;16: 1-26.
165. Hindáková M & Záborský J. In: Löve Á, editor. IOPB chromosome number reports. LVI. Taxon. 1974;26: 257-274.
166. Lovka M, Sušnik F, Löve Á & Löve D. In: Löve Á, editor. IOPB chromosome number reports. XXXVI. Taxon. 1972;21: 333-346.
167. Bosemark NO. Further studies on accessory chromosomes in grasses. Hereditas. 1957;43: 236-298.
168. Lövkvist B. In: Weimarck H, editor. Skånes flora. Lund: Corona; 1963.
169. Heiser CB & Whitaker TW. Chromosome number, polyploidy and growth habit in California weeds. Amer J Bot. 1948;35: 179-186.
170. Mehra PN & Sharma ML. In: Löve Á, editor. IOPB chromosome number reports. XXXIX. Taxon. 1973;22: 115-118.
171. Teppner H. *Anthoxanthum maderense* spec. nova und *A. odoratum* (Poaceae-Aveneae) von Madeira und deren chromosomen morphologie. Phyton (Horn). 1998;38: 307-321.
172. Valdès B. Revisión de las especies anuales del género *Anthoxanthum* (Graminae). Lagascalia. 1973;3: 99-141.

173. Östergren G. Heterochromatic B-chromosomes in *Anthoxanthum*. Hereditas. 1947;33: 261-296.

174. De Leonardis W, Pavone P, Terrasi MC, Zizza A. Numeri cromosomici per la Flora Italiana: 814-830. Inform Bot Ital. 1981;13: 158-167.
